# Wrinkle-Improving Effect of Novel Peptide That Binds to Nicotinic Acetylcholine Receptor

**DOI:** 10.3390/ijms25147860

**Published:** 2024-07-18

**Authors:** Jinho Bang, Yul-Lye Hwang, Mi Yoon Kim, Jae Nam Yun, Eujin Hyun, Min Youl Chang, Dae Hwan Shin, Sunghyun Kim, Jeung-Hoon Lee

**Affiliations:** 1 SKINMED R&D Center, Daejeon 34037, Republic of Korea; bjh619@skinmed.biz (J.B.); hwangyull@skinmed.biz (Y.-L.H.); miyoonkim@skinmed.biz (M.Y.K.); woska101@skinmed.biz (J.N.Y.); eujinhyun@skinmed.biz (E.H.); 2 College of Pharmacy, Chungbuk National University, Cheongju 28160, Republic of Korea; dshin@chungbuk.ac.kr; 3 SKINMED Clinical Trials Center, Daejeon 34050, Republic of Korea; mychang195@google.com; 4 Bio-Healthcare Materials Center, Korea Institute of Ceramic Engineering and Technology, Cheongju 28160, Republic of Korea

**Keywords:** acetylcholine, nicotinic acetylcholine receptor, collagen, aquaporin, hyaluronic acid, wrinkle, clinical efficacy, peptide

## Abstract

Wrinkles, one of the most common signs of aging, are primarily caused by the continuous contraction of muscles. Muscle contraction is induced by the binding of acetylcholine (ACh), released at the neuromuscular junction, to nicotinic acetylcholine receptor (nAChR) present on the muscle cell surface. In this study, we aimed to develop a wrinkle-improving peptide that inhibits the binding of ACh to nAChR using peptide phage display technology. Our peptide showed a remarkably high binding affinity to nAChR subunit α1, with a value below 1 µM, and was found to inhibit the action of ACh through its interaction with these receptors. Furthermore, it increased collagen synthesis in skin cells and upregulated the expression of the aquaporin-3 (AQP3) and hyaluronan synthase-2 (HAS2) genes. These results confirm that the peptide effectively inhibits muscle contraction and enhances skin elasticity and hydration, contributing to its wrinkle-reducing effects. Clinical studies on humans observed significant improvement in wrinkles after three weeks of use, with substantial reduction observed after six weeks. In conclusion, these findings demonstrate the efficacy of the peptide (named Medipep) in reducing wrinkles.

## 1. Introduction

As skin ages, it undergoes numerous changes, with facial wrinkles being one of the most prominent effects [1]. The causes of facial wrinkles include a combination of factors such as UV exposure, oxidative damage, and heat, leading to the degradation of matrix proteins such as elastin fibers and a decrease in moisture content [2,3,4]. Additionally, wrinkles are formed and become more pronounced over time due to the continuous contraction of facial muscles [5,6].

Muscle contraction is induced by acetylcholine (ACh), a neurotransmitter released from nerve cells [7]. ACh is released into the space between muscles and nerves when synaptic vesicles fuse with the cell membrane through the action of the SNARE (Soluble N-ethylmaleimide-sensitive factor attachment protein receptor) complex within nerve cells. Released ACh binds to nAChR or muscarinic acetylcholine receptor (mAChR) on the muscle cell surface to induce muscle contraction [8,9]. Facial wrinkles are formed by muscle contraction through the binding of muscle nAChRs (subtypes α1, β1, δ, γ, and ε) present on the skeletal muscle cell surface [10,11,12]. These facial wrinkles can be effectively improved through muscle relaxation by inhibiting the formation of SNARE complexes to inhibit ACh release, or by binding to nAChRs instead of ACh to inhibit the action of ACh. Utilizing these methods, cosmetic peptides such as Argireline^®^ (Lipotec, Spain) and SYN^®^-AKE (DSM, Netherlands) are currently widely used for wrinkle improvement [13,14]. According to previous research results, Argireline^®^ is a peptide that binds to the SNARE complex and inhibits ACh secretion, and the wrinkle reduction effect is known to be about 30% [15]. SYN^®^-AKE, which binds to nAChRs and inhibits the action of ACh, has a reported IC_50_ of 180 μM [16], and both ingredients have been proven to be effective in improving wrinkles.

Therefore, this study aimed to develop a more efficient novel peptide that produces a potential anti-aging effect while reducing facial skin wrinkles by inhibiting muscle contraction. We screened for peptides specific to the nAChR subunit α1 using a peptide phage display technique. After several rounds of enrichment, ELISA was performed to confirm the sequence of the selected peptides, and then their binding affinity to the nAChR subunit α1 was measured using surface plasmon resonance (SPR). Through a process of verifying the inhibition of the nicotine response in a concentration-dependent manner using cells expressing nAChRs, it was confirmed whether the peptides could bind to nAChRs and inhibit the action of ACh. We also evaluated the impact of these peptides on collagen protein synthesis affecting skin elasticity and examined the expression of factors influencing skin hydration, HAS2, and AQP3. Finally, the safety of the peptides was confirmed by evaluating skin irritability and eye irritability, and the anti-aging effects of the peptides were verified through clinical efficacy tests on facial wrinkles.

## 2. Results

### 2.1. The Screening of Phage Peptides That Specifically Bind to the Surface of nAChR Subunit α1

**Figure 1:**
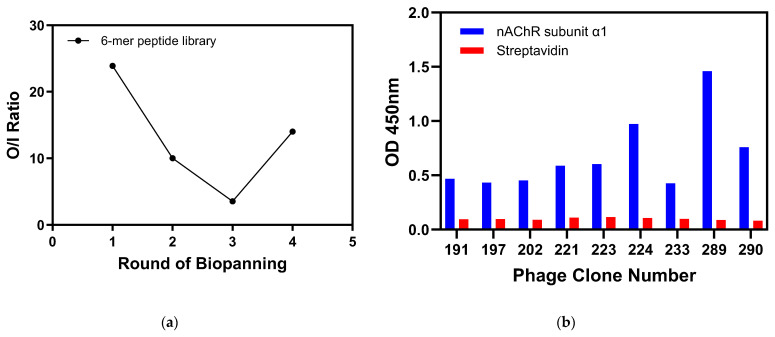
Biopanning for nAChR subunit α1-specific peptides. (**a**) Measurement of output/input phage (O/I) ratio in all rounds of biopanning. (**b**) ELISA of phage clones in binding to nAChR subunit α1 compared to streptavidin as negative control.

In this study, we first screened and identified peptides that could specifically bind to the surface of nAChR subunit α1 using the 6-mer phage display peptide library. The output/input phage ratio was calculated for each round of nAChR subunit α1 biopanning. The ratio was decreased until Round 3 and then increased during the fourth round (Figure 1a). The data showed that the number of fourth-round phages was 3.94 times higher than that of the third-round experiment (Table 1). The 310 phage clones from the fourth round of biopanning underwent ELISA to select the nAChR subunit α1 binding phage clones. As shown in Figure 1b, nine phage clones had a very high binding affinity to the nAChR subunit α1 compared to that of streptavidin. Thus, these nine positive phage clones were propagated, and their DNA was sequenced to confirm the amino acid sequences of the peptides (Table 2 and Table S1).

### 2.2. The Binding Affinity of the Phage Display-Derived Peptides with the nAChR Subunit α1

**Figure 2:**
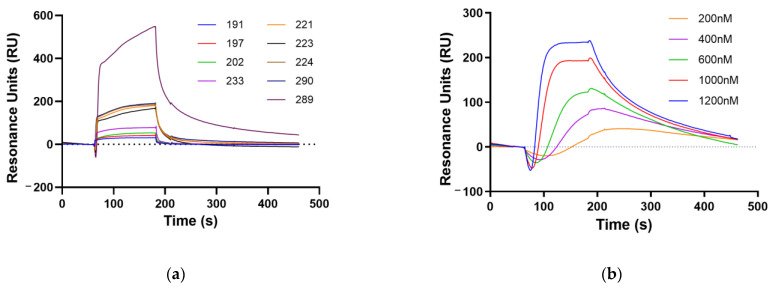
Binding affinities of synthesized phage display-derived peptides. (**a**) Binding analysis of nine synthesized peptides at same concentration (5 μM) to nAChR subunit α1 using SPR. (**b**) Analysis of binding affinity to nAChR subunit α1 using different concentrations of peptide 289 (200, 400, 600, 1000, and 1200 nM).

We performed an SPR analysis to confirm the binding affinities of the nine peptides to nAChR subunit α1. The nAChR subunit α1 was immobilized on a sensor chip, and we then tested the nine peptides with the same concentration (5 μM) using SPR analysis and found that peptide 289 had the highest amount of resonance units (RU) compared to the other peptides (Figure 2a). These data show that peptide 289 has a stronger binding affinity to nAChR subunit α1 than the other peptides. Based on these results, we selected peptide 289 as a novel peptide (named Medipep). To measure the binding affinity of peptide 289 to nAChR subunit α1, SPR analysis was performed at different concentrations (200, 400, 600, 1000, and 1200 nM) (Figure 2b). According to the results of kinetic analysis, peptide 289 showed an association rate constant (ka) of 1.49 × 10^4^ M^−1^ s^−1^ and a dissociation rate constant (kd) of 9.06 × 10^−3^ s^−1^, yielding an equilibrium dissociation constant (KD) of 6.09 × 10^−7^ M (Table 3).

### 2.3. The Effect of a Novel Peptide on Inhibiting the Action of ACh by nAChR Interaction

**Figure 3:**
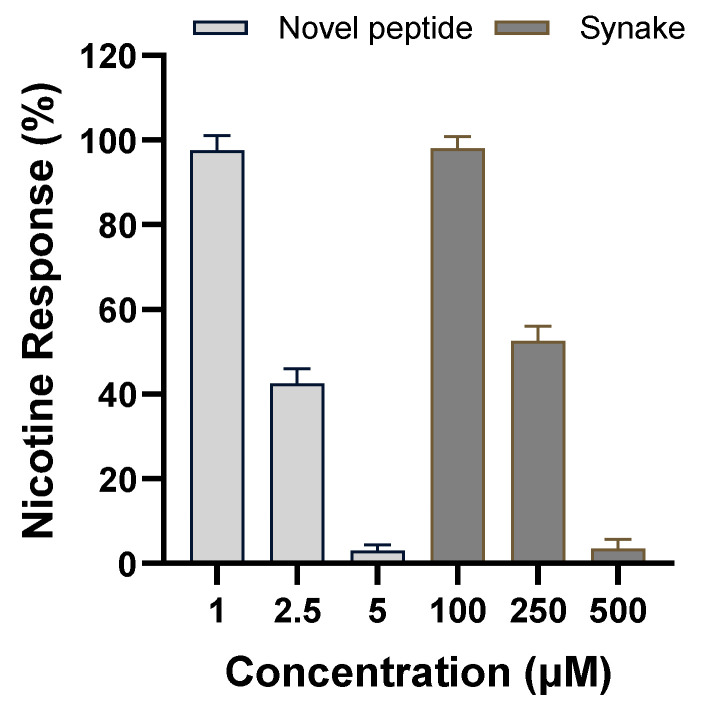
The nicotine response analysis for nAChRs with our novel peptide (Medipep) and SYN^®^-AKE as a positive control. The novel peptide and SYN^®^-AKE were treated at different concentrations and then the response to 400 μM nicotine was confirmed. The results are presented as the mean ± SE of three repeated measurements.

We evaluated whether the peptide could bind to muscle nAChR and inhibit the action of ACh. For the evaluation, we used nicotine, which binds to the same nAChR site as ACh, instead of ACh, which binds to both mAChRs and nAChRs. Nicotine acts as an agonist in the same way as ACh [17]. As shown in Figure 3, our peptides showed nicotine responses of 97%, 42.5%, and 3% at concentrations of 1, 2, and 5 μM, respectively. In addition, the positive control, SYN^®^-AKE, showed nicotine responses of 98%, 52.5%, and 3.5% at concentrations of 100, 250, and 500 μM. That is, our peptide showed a similar nicotine inhibitory effect even at about 1/100 of the positive control’s concentration, and we confirmed that the nicotine response was inhibited in a concentration-dependent manner. Since nicotine acts as an agonist in the same way as ACh, these results suggest that the peptide can inhibit the action of ACh by binding to nAChRs.

### 2.4. The Effects on Collagen Production and AQP3/HAS2 Gene Expression by a Novel Peptide

**Figure 4:**
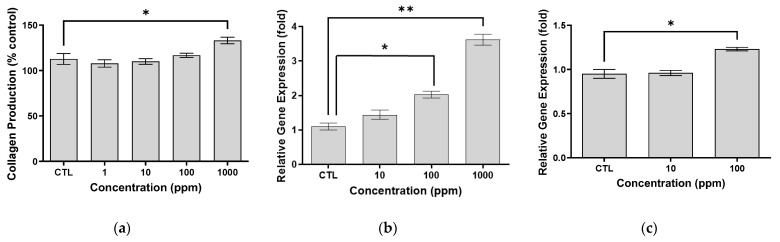
Stimulation of collagen production in cultured dermal fibroblasts, and expressions of AQP3 and HAS2 in keratinocyte in vitro. (**a**) Increased production of collagen by our peptide was observed in cultured human dermal fibroblasts by enzyme-linked immunosorbent assay (ELISA). mRNA expression of (**b**) AQP3 (**c**) and HAS2 in keratinocytes treated with our peptide (*: *p* < 0.05; **: *p* < 0.01).

To evaluate the effects of our novel peptide (Medipep) on collagen expression, enzyme-linked immunosorbent assay (ELISA) analysis was performed on dermal fibroblasts. As a result, collagen expression slightly increased at 1000 ppm, and the extent was statistically significant (an approximate 1.18-fold increase in 1000 ppm of the peptide) (Figure 4a). In addition, to confirm the expressions of the aquaporin-3 (AQP3) and hyaluronan synthase-2 (HAS2) genes in the treated keratinocytes, in vitro real-time PCR was performed. As shown in Figure 4b,c, significant increases in AQP3 and HAS2 gene expression were observed. Therefore, we confirmed that the peptide affects increased collagen production as well as AQP3 and HAS2 gene expression.

### 2.5. Safety Evaluation of Novel Peptide: Cytotoxicity, Skin Irritation, and Eye Irritation

Prior to carrying out the clinical trial, cytotoxicity, skin irritation, and eye irritation were evaluated to confirm the safety of the peptide. During the experiment, the peptide showed no toxicity at a concentration of 0.1 ppm to 100 ppm (Figure S1). Skin reactivity, derived from human patch testing in 31 subjects, is summarized in Table S2. The results indicated a skin reactivity score of 0.0 ± 0.00 for a novel peptide concentration ranging from 0.1 ppm to 100 ppm, signifying non-irritation in accordance with the criteria outlined in the Frosch and Kligman and COLIPA guidelines. In addition, the eye irritation potential of the peptide was evaluated using the MCTT HCE^TM^ evaluation method using a human cornea model, and it was found to be non-irritating in the concentration range of 0.1 ppm to 100 ppm (Table S3).

### 2.6. Anti-Wrinkle Efficacy of Novel Peptide in Clinical Study

**Figure 5:**
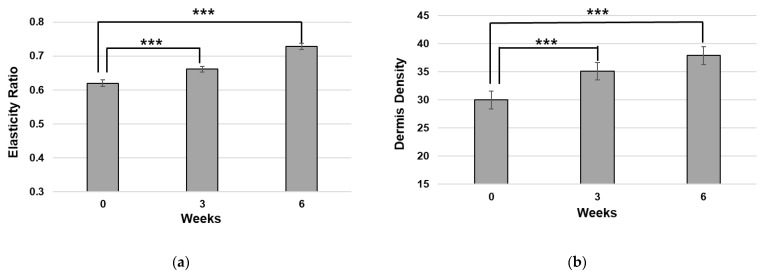
The novel peptide treatment increases skin elasticity and dermal density. (**a**) Skin elasticity, measured using a Dermal Torque Meter, and (**b**) dermal density, measured using the ultrasound device DUB Skinscanner, were significantly improved (***: *p* < 0.001).

To further evaluate the anti-aging efficacy of the peptide, a clinical study was performed on normal, healthy volunteers. A total of 20 female volunteers with a mean age (±standard deviation (SD)) of 49.4 (±5.08) (min. 40; max. 65) completed the study, wherein the elasticity of each subject’s cheek area, density of the dermis, and crow’s feet wrinkles were measured. After three weeks and six weeks of usage, measured elasticity and dermis density were significantly improved compared to the baseline value (Figure 5a,b).

**Figure 6:**
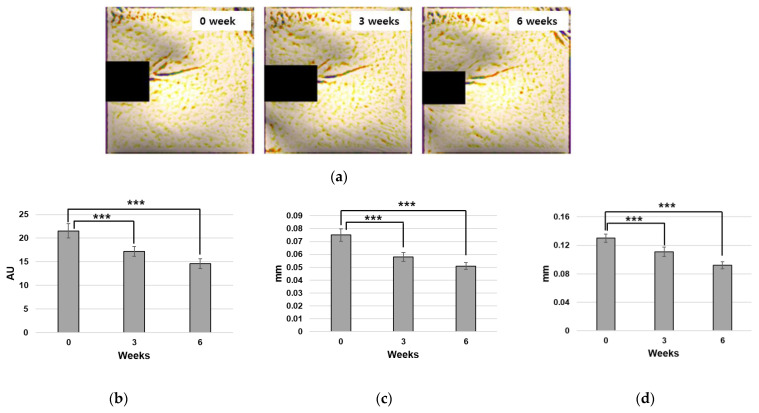
Reduction of skin wrinkles by novel peptide treatment. (**a**) Representative images of Antera 3D photographs after image processing. Significant improvements of skin wrinkles were observed in (**b**) overall size, (**c**) depth, and (**d**) maximum depth of crow’s feet (***: *p* < 0.001).

The evaluation revealed notable improvements in various parameters associated with crow’s feet wrinkles. Specifically, the overall size, depth, and maximum depth of crow’s feet wrinkles exhibited statistically significant reductions at both the 3rd and 6th weeks post-product application compared to the baseline measurements (Figure 6). In detail, there was a significant decrease in overall size of 31.5%, a reduction in depth of 30.1%, and a noteworthy decline in maximum depth of 27.9% (Table 4).

## 3. Discussion

As the skin ages, it undergoes gradual morphological and physiological declines, exhibiting clear signs of aging [18,19]. Skin aging can be classified into two types: intrinsic aging, a natural phenomenon dictated by genetics as we age, and extrinsic aging, which occurs due to external environmental factors, predominantly caused by UV exposure [20]. The combination of intrinsic and extrinsic factors leads to the characteristic indicator of aged skin: wrinkles [21]. Wrinkles arise from a variety of causes, including damage to matrix proteins such as elastin fibers; dehydration; muscle movement in the face; UV exposure; oxidative damage; and heat [19,22,23,24].

This study focused primarily on the movement of facial muscles as a cause of wrinkle formation. Facial muscle movement, induced by the neurotransmitter ACh released from nerve cells, involves the fusion of synaptic vesicles mediated by the SNARE complex and the subsequent release of ACh into the synapse between muscle and nerve [8,25]. The released AChs bind to nAChRs on muscle cells, causing contraction [26]. Repeated muscle contraction over time leads to the formation and deepening of wrinkles [27]. In pursuit of potential anti-aging effects such as reducing facial skin wrinkles, this study aimed to develop a novel peptide that inhibits muscle contractions.

Phage display technology is widely used and is a useful method for finding new peptides [28]. This technique involves inserting a peptide coding gene into a phage gene to create a library of billions of peptides, allowing for the simultaneous screening of up to a billion different peptides against a desired target [29,30]. We set the target binding site to the nAChR subunit α1 and performed the phage display. Clones were collected through a total of four enrichment processes, and each clone was measured by ELISA, after which nine clones were selected in order of high specific binding affinity to the nAChR subunit α1. SPR analysis was used to measure the binding force between the nine peptides and the nAChR subunit α1. SPR analysis is a general method used to measure the binding force between a peptide and a receptor using association and dissociation rates [31,32]. The best peptide (peptide 289, Medipep) was selected by measuring the SPR of each of the nine peptides, and the binding affinity of the best peptide to the nAChR subunit α1 was 609 nM.

In previous studies, ACh has been shown to bind to the C-loop of subunit α1, the allosteric site of the nAChR [33,34,35,36]. Based on this, we treated TE671 cells expressing nAChRs with our peptide and nicotine (which acts as an agonist in the same way as ACh), and it was confirmed that the nicotine response was inhibited in a concentration-dependent manner [37]. The results showed that while the SYN^®^-AKE peptide required a very high concentration of 500 µM to inhibit the nicotinic response, our peptide demonstrated similar inhibition at a concentration approximately 1/100 of that. This allows us to hypothesize that our peptide binds to the C-loop of subunit α1, the allosteric site of nAChR, thereby inhibiting ACh from binding to the receptor and attenuating wrinkle formation by muscle contraction. However, to prove this hypothesis, we believe that it will be necessary to identify the exact mechanism through three-dimensional structural modeling during further study.

The formation of wrinkles due to facial muscle movement is more pronounced in skin that is dry and less elastic, highlighting the importance of maintaining skin elasticity and hydration [38,39]. Collagen, along with elastin, forms the structural support that maintains the strength and elasticity of the skin and is one of the basic components of the extracellular matrix (ECM) in the skin [40]. Collagen loss leads to degeneration of the skin’s support structure, reducing its elasticity [41]. Additionally, hyaluronic acid, synthesized by the enzyme HAS2, fills the space between collagen and elastin fibers in the dermis, storing moisture to hydrate and protect the skin [42,43]. Aquaporins—water channel proteins on the cell membrane—primarily regulate the movement of water between the inside and outside of cells, with AQP3 mainly present in the skin, maintaining hydration [44,45,46].

Therefore, when we evaluated the peptide’s effects on collagen protein synthesis and on HAS2 and AQP3 gene expression in the skin, we found that collagen synthesis and gene expression were significantly increased. Additionally, in vitro fibroblast testing confirmed that the peptide was non-cytotoxic up to concentrations of 100 μg/mL. Additionally, the safety of the peptide was confirmed through eye and skin irritation tests. Based on these results, a human clinical trial showed significant improvement in wrinkles after 3 weeks of use and a noticeable reduction in skin wrinkles after 6 weeks, with a decrease in overall size of 31.5%, a reduction in depth of 30.1%, and a noteworthy decline in maximum depth of 27.9%.

In conclusion, the results of this study demonstrate that the peptide (named Medipep) produces a wrinkle-improving effect by controlling factors related to muscle contraction, skin elasticity, and moisture, confirming its anti-aging effect in clinical trials.

## 4. Materials and Methods

### 4.1. Materials and Reagents

The ligand used recombinant human acetylcholine receptor subunit α1, Cat. No. MBS963713 (MyBioSource, San Diego, CA, USA), and SYN^®^-AKE used dipeptide diaminobutyroyl benzylamide (Cayman Chemical, Ann Arbor, MI, USA). All peptides used in this study were synthesized and supplied by Anygen (Gwangju, Republic of Korea). Phosphate-buffered saline (PBS), Tween 20, polyethylene glycol (weight-averaged molecular weight (MW) = 8000), LB agar, and bovine serum albumin (BSA) were purchased from Sigma-Aldrich (St. Louis, MO, USA). All reagents and cell culture media were purchased from WELGENE (Gyeongsan-si, Republic of Korea). The INCI name for the novel peptide is Palmitoyl Hexapeptide-93. It was synthesized by Incospharm (Daejeon, Republic of Korea), and clinical trials were conducted with a content of 500 ppm *(w/v)*.

### 4.2. Cell Culture

TE671 rhabdomyosarcoma cell line was purchased from Korea Cell Line Bank (Seoul, Republic of Korea). Dermal fibroblasts (Hs68) were purchased from American Type Culture Collection. Cells were cultured in Dulbecco’s Modified Eagle’s medium (DMEM) and medium containing 10% fetal bovine serum (FBS), 1% penicillin-streptomycin at 37 °C, and up to 5% CO_2_. Human skin keratinocytes (HEKn) were purchased from Thermo Fisher Scientific (Waltham, MA, USA) and cultured using keratinocyte growth medium, keratinocyte-SFM supplemented with epidermal growth factor (EGF) and bovine pituitary extract (BPE) at 37 °C, and up to 5% CO_2_.

### 4.3. Phage Library and Biopanning

**Figure 7:**
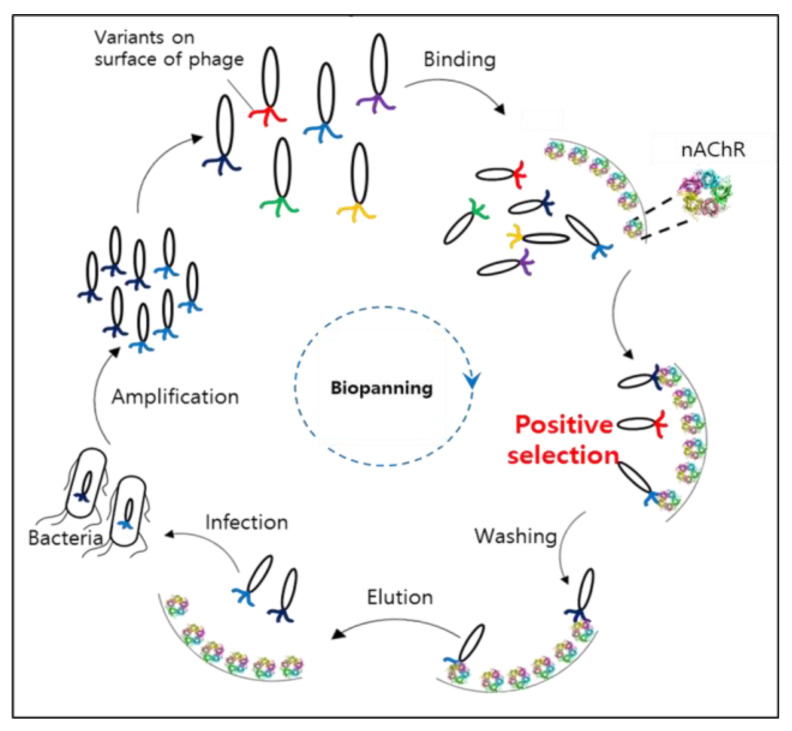
Schematic of the 6-mer phage display peptide library.

The biopanning technique was designed in a previous study and used with some modifications [28]. It is shown in Figure 7 as an affinity selection technology used to identify peptides that bind to a given target. The 6-mer phage display peptide library was constructed through NNK codon-based randomization (N = A or C or G or T; K = G or T). The gene fragments were double digested with *SfiI/NotI* and cloned into pIGT2 phagemid vectors. The resulting constructs were transformed into *Escherichia coli* (*E. coli*) cells and the naive library of 6-mer peptides was composed of 1.23 × 10^7^ independent 6-mer peptide clones. The 6-mer peptide phage library was prepared using the M13KO7 helper phage. The nAChR subunit α1 was used as the target protein during phage selection. The nAChR subunit α1 was immobilized directly on 96-well high-binding plates overnight at 4 °C. After that, the plates were incubated in blocking buffer (PBS containing 2% BSA) for 2 h at room temperature. The prepared 6-mer peptide phage was added to the plates for 1 h at 30 °C. Unbound phages were removed by washing with PBS containing 0.05% Tween 20 (three times in rounds 1 and 2, four times in rounds 3 and 4). Bound phages were subsequently eluted by incubation with 0.2 M glycine HCl (pH 2.2) for 10 min, followed by immediate neutralization with 1 M Tris HCl (pH 9.0). To perform the next round of biopanning, the eluted phages were used to infect *E. coli* for 1 h at 37 °C, and helper phages were then added and incubated for 18 h at 37 °C. The culture was then centrifuged at 12,000× *g* for 10 min at 4 °C. After that, the supernatant was transferred into a new tube with the addition of one-sixth volume of 20% polyethylene glycol (PEG)/2.5 M NaCl and incubated for 1 h at 4 °C. The PEG-precipitated phages were then centrifuged at 12,000× *g* for 1 h at 4 °C. The phage pellet was resuspended in 1000 μL of PBS, and that was the first nAChR subunit α1-specific sublibrary. At each step, the input and output phage PFU in all the rounds of biopanning were measured.

### 4.4. Enzyme-Linked Immunosorbent Assay (ELISA)

To identify phage clones, we performed an ELISA assay. After four rounds of in vitro biopanning, 310 were randomly chosen from the titration plate. For this purpose, 5 μg/mL of nAChR subunit α1 was used to coat a Nunc MaxiSorp 96-well plate and left at 4 °C overnight. After blocking with 2% BSA, the plate was washed three times with PBS-T (PBS with 0.05% *v*/*v* Tween 20), and the supernatants from the phage culture were applied to the plates and incubated for 1 h at 37 °C. After washing three times, the HRP-conjugated anti-M13 antibody (1:1000 in BSA) was incubated on the plate for 1 h. The plate was washed three times to get rid of the nonspecific antibody, followed by color development with the addition of the 3,3′,5,5′-tetramethylbenzidine (TMB) substrate. The incubation was stopped by adding 2 M H_2_SO_4_. Finally, the absorbance was measured at 450 nm using a microplate reader. The positive clones were subjected to DNA sequencing using a phagemid primer (5′-GATTACGCCAAGCTTTGGAGC-3′; Bioneer, Daejeon, Republic of Korea).

### 4.5. Binding Affinity Using Surface Plasmon Resonance (SPR) Assay

The SPR analysis was performed using a BIACORE 3000 (GE healthcare, Chicago, IL, USA). The nAChR subunit α1 was immobilized on a CM5 sensor chip by injecting the protein diluted in PBS (0.2 M phosphate buffer, 0.027 M KCl, 1.37 M NaCl, pH 7.4) at a concentration of 0.35 mg/mL at a flow rate of 5 μL min^−1^. After, 5 μM peptide was injected into running buffer (20 mM Tris, pH 7) and its interaction with nAChR subunit α1 was measured at a flow rate of 30 μL min^−1^. For kinetic experiments, peptides diluted in running buffer at concentrations ranging from 100 μL to 200, 400, 600, 1000, and 1200 nM were injected at a flow rate of 30 μL min^−1^ over the immobilized nAChR subunit α1. The association and dissociation kinetic rate constants (ka and kd) and the equilibrium association constant, KD, were calculated using BIAevaluation 3.1 software [47].

### 4.6. In Vitro Intracellular Nicotine Response Assay

TE671 cells were cultured in DMEM and medium containing 10% FBS, 1% penicillin-streptomycin at 37 °C, and up to 5% CO_2_. An 18 mm coverslip was placed on a 12-well cell culture plate, cultured cells were removed with trypsin, and 1 mL was dispensed into each well to make 2 × 10^4^ cells/well, followed by culturing for 4 days. The coverslip with the cultured cells was transferred to a new 12-well plate, mixed with 997 μL of HBSS buffer and 3 μL of Fura-2-AM (Invitrogen, Waltham, MA, USA), and incubated for 15 min. After incubation, the remaining Fura-2-AM was removed by washing three times with HBSS buffer, and 1 mL of HBSS buffer was additionally dispensed. After mounting the coverslip in the chamber (Live Cell Instrument, Namyangju-si, Republic of Korea), 500 μL of HBSS buffer was dispensed, and the peptide and nicotine samples to be tested were adjusted according to the concentration. Then, 10 to 20 cells were selected and captured alternately at 340 nm and 380 nm wavelengths at 500 ms intervals through a fluorescence microscope (DMI3000 B, Leica Microsystems, Wetzlar, Germany). The fluorescence ratio at 340/380 nm was calculated using the LAS X program [48].

### 4.7. In Vitro Cell Viability Assay

The in vitro cytotoxicity of the novel peptide on cells was assessed using the MTT [(3-(4,5-dimethylthiazol-2-yl)-2,5-diphenyltetrazolium bromide] cell viability assay. Briefly, dermal fibroblasts were cultured in 24-well plates at a density of 1 × 10^5^ cells/well for 18 h. After the medium was removed, the sample diluted in FBS-free medium was added and cultured for 24 h. Then, after the medium was removed, MTT solution was added at a concentration of 1 μg/mL and left to react for 3 h. Unreacted MTT was removed, 100 μL of DMSO was added to dissolve the formed reaction product, and the absorbance at 540 nm was measured and evaluated using an ELISA reader.

### 4.8. Measurement of Collagen Production and Gene Expression

Dermal fibroblasts were cultured in 48-well plates at a density of 2~5 × 10^4^ cells/well for 24 h. The existing medium was removed, and the medium containing the sample was added. The culture was incubated for 24 h, after which the cell culture medium was collected and subjected to ELISA assay. The Human Pro-Collagen α1 DuoSet^®^ ELISA kit used in this test was from R&D Systems (Minneapolis, MN, USA) and was used according to the experimental protocol provided by the manufacturer.

For sample processing, keratinocytes were seeded in a 60 mm culture plate at a concentration of 2~5 × 10^5^ cells/mL and cultured for 24 h. The growth medium was removed, and the samples were added at different concentrations to keratinocyte-SFM without EGF and BPE, after which the keratinocytes were treated for 24 h. Total RNA was isolated from cultured epidermal keratinocytes using an Easy-blue RNA Extraction Kit, Cat. No. 17061 (iNtRON Biotechnology, Seongnam-si, Republic of Korea). All primers for the quantitative reverse transcription PCR (qRT-PCR) (Table S4) were designed using Primer Express v.1.5 (Applied Biosystems, Waltham, MA, USA) software. Total RNA (2 μg) was reverse transcribed to complementary DNA with an M-MLV reverse transcriptase. The resulting complementary DNA was amplified using a SYBR Green Master Mix kit, Cat. No.EBT-1802 (ELPIS Biotech, Daejeon, Republic of Korea). Real-time quantitative reverse transcription polymerase chain reaction (qRT-PCR) was performed using an AB StepOne Real-Time PCR system (Applied Biosystems, USA). All samples were analyzed using the comparative C_t_ method (2^−ΔΔCt^) for the relative quantification of gene expression and normalization with respect to glyceraldehyde 3-phosphate dehydrogenase (GAPDH) expression.

### 4.9. Skin Irritation Test

The skin irritation test was conducted at CRA Korea Co., Ltd. in the Republic of Korea, with the approval serial number CRA21-HIPTRP1001. The patch used was the IQ Ultra Chamber (Chemotechnique Diagnostics, Vellinge, Sweden). The human skin primary irritation test was completed, with all 31 subjects participating until the end of the study. The test area for the product was the back (excluding the spinal area). The patch was applied for 24 h and then removed. The first evaluation was conducted 30 min after patch removal while waiting for the transient erythema to disappear due to removal, and the second evaluation was conducted 24 h after patch removal. The irritation criterion used a measurement evaluation method designed by Frosch and Kligman by applying the reading standards of the International Contact Dermatitis Research Group (ICDRG).

### 4.10. Eye Irritation Test

To evaluate the eye irritation potential of the peptide, an in vitro experimental model recognized by the OECD as an alternative animal evaluation method, the human cornea model (MCTT HCE^TM^), was used. The experiment was conducted according to the standard experimental method provided by the manufacturer, Biosolution (Seoul, Republic of Korea). First, before the experiment, the color interference of the test material was checked, and to begin the experiment, MCTT HCE^TM^ was incubated for 22 h at 5% CO_2_ and 37 °C. Next, 40 μL of the peptide was applied to the top of the MCTT HCE^TM^ and left to react for 10 min. Afterwards, the peptide was removed using DPBS, and MCTT HCE^TM^ was transferred to a new medium and cultured for 18 h at 5% CO_2_ and 37 °C. After the culture was completed, 200 μL/well of WST-1 solution diluted at a ratio of 1:25 was added to a new plate, MCTT HCE^TM^ was transferred thereon, and 100 μL of WST-1 solution was treated inside. Finally, after blocking the light with foil and culturing for 3 h at 5% CO_2_ and 37 °C, the WST-1 solution inside and outside the tissue was collected and transferred to a 96-well plate, and the absorbance was measured at 450 nm.

### 4.11. Clinical Study

The open-label clinical trial (CRA21-CT1400) was conducted on 20 female Asian subjects with facial wrinkles, aged 40 to 65 years old, and the trial was approved by the Institutional Review Board of CRA Korea Co., Ltd. (15 September 2021). All studies complied with the World Medical Association’s Declaration of Helsinki concerning biomedical research involving human subjects. Healthy female volunteers without any skin or systemic diseases were initially enrolled in the study. Subjects who had been treated with retinoids or laser therapy within the last six months, or who had participated in another clinical study, were excluded. After hearing the explanation of the purpose and protocol of the study, all the volunteers signed the informed consent form and participated in the study. A total of 21 female volunteers initially enrolled in the study, but during the study, 1 participant was dropped due to failure to attend the scheduled visit, and 20 participants completed the study. During the first visit, subjects were asked to complete study-related medical record questionnaires to confirm the inclusion and exclusion criteria, and each participant provided written, informed consent. Before the instrumental measurements, participants were asked to rest for at least 30 min in a humidity (50 ± 10% RH)- and temperature (22 ± 2 °C)-controlled room. The torsional elasticity and dermal density of the skin in each subject’s cheek area were measured using a Dermal Torque Meter (DTM310, Dia-Stron, Andover, UK) and an ultrasound device, the DUB Skinscanner (tpm, Lueneburg, Germany) [49]. Wrinkles around the eyes were measured using a topographic skin measurement device (Antera 3D CS: Miravex Limited, Dublin, Ireland) [50]. The participants were administered with 619.9 ± 241.19 mg of essence containing the novel peptide twice a day (morning and noon) for 6 weeks. The concentration of the peptide was 500 ppm, determined based on formulation studies at levels that were found to be non-irritating and non-allergenic during preliminary testing. After using the product, they revisited the clinical institution in the 3rd and 6th weeks, where the same measurements as those taken before product use (week 0) were conducted. Subsequently, the results from before and after product usage were compared and evaluated.

### 4.12. Statistical Analysis

Since the number of subjects was lower than 30, a normality test was performed using the Shapiro–Wilk test to properly analyze the experimental results. If normality was satisfied, a parametric test was performed, and if normality was not satisfied, statistical analysis was performed using a non-parametric test. The measurements of skin moisture content, wrinkles around the eyes (overall size), and dermal density before and after using the product were compared using the paired *t*-test, which is a parametric test, and the Wilcoxon signed-rank test, which is a non-parametric test, to obtain the size results of wrinkles around the eyes (depth/maximum depth). The statistical analysis results were checked for significance with a 95% confidence interval, and IBM SPSS Statistics software, version 28.0 was used as the statistical analysis program.

## 5. Conclusions

In this study, we developed a novel peptide that simultaneously regulates muscle contraction, skin elasticity, and moisture, and investigated its specificity and anti-aging effect in vitro. The clinical efficacy of the peptide (named Medipep) as an anti-wrinkle cosmetic ingredient was also proven.

## Figures and Tables

**Table 1:** Specific enrichment of nAChR subunit α1-bound phages using initial input of 9.6 × 10^10^ PFU (plaque-forming units).

Round	Inputs (PFU/mL)	Outputs (PFU/mL)	Output phage/Input Phage Ratio
1	9.6 × 10^10^	2.3 × 10^7^	23.9 × 10^−5^
2	1.98 × 10^12^	1.98 × 10^8^	10 × 10^−5^
3	1.63 × 10^12^	5.8 × 10^7^	3.55 × 10^−5^
4	3.77 × 10^12^	5.28 × 10^8^	14 × 10^−5^

**Table 2:** Specific peptides for nAChR subunit α1.

Peptide Number	Sequence	nAChR Subunit α1 (OD_450_)	Streptavidin (OD_450_)	nAChR Subunit α1/Streptavidin Ratio
289	RRGVRR	1.46	0.088	16.59091
290	RKRIRR	0.757	0.081	9.345679
224	KRGRCK	0.973	0.106	9.179245
221	RKRQTR	0.587	0.109	5.385321
223	KRRFQK	0.603	0.114	5.289474
202	KRQWVK	0.453	0.09	5.033333
191	RRTSWR	0.468	0.094	4.978723
197	KRLQAR	0.432	0.096	4.5
233	RRQTHK	0.424	0.097	4.3711134

**Table 3:** Binding affinity data of novel peptide (peptide 289, Medipep) to nAChR subunit α1 using SPR.

ka (1/Ms)	kd (1/s)	Rmax (RU)	KA (1/M)	KD (M)
1.49 × 10^4^	9.06 × 10^−3^	369	1.64 × 10^6^	6.09 × 10^−7^

ka: association constant; kd: dissociation constant; Rmax: maximum binding capacity; KA: equilibrium association constant; KD: equilibrium dissociation constant/affinity.

**Table 4:** The clinical efficacy of the novel peptide. Significant improvement of overall size, depth, and maximum depth were confirmed by image analysis using an Antera 3D camera (Miravex Limited, Dublin, Ireland).

Measurement	Weeks	% Improvement (Mean ± SD)	*p*-Value
Overall size (AU)	0	-	-
	3	19.1 ± 7.84	<0.001
	6	31.5 ± 9.18	<0.001
Depth (mm)	0	-	-
	3	21 ± 9.18	<0.001
	6	30.1 ± 10.27	<0.001
Maximum depth (mm)	0	-	-
	3	14.2 ± 16.79	<0.004
	6	27.9 ± 16.41	<0.001

AU: arbitrary units.

## Data Availability

Data is contained within the article and Supplementary Materials.

## Supplementary Materials

The following supporting information can be downloaded at: https://www.mdpi.com/article/10.3390/ijms25147860/s1.
