# Supplementary material for: Wrinkle-Improving Effect of Novel Peptide That Binds to Nicotinic Acetylcholine Receptor
Source: Int J Mol Sci. 2024 Jul 18;25(14):7860. doi: 10.3390/ijms25147860 (PMC11277145; doi:10.3390/ijms25147860)

# Supplementary Material

Supplementary Figure S1. Viability of dermal fibroblast cells treated with a novel peptide (peptide 289, Medipep)

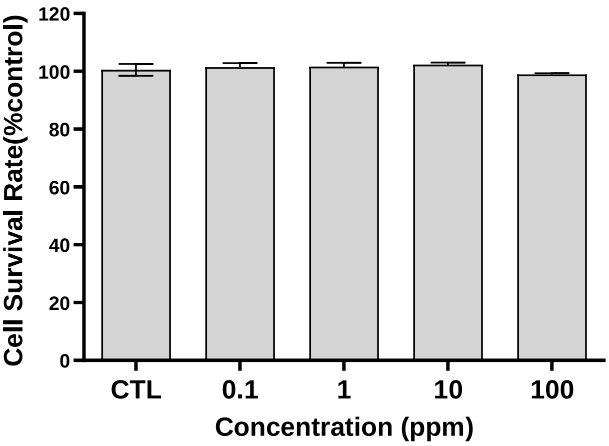


Supplementary Table S1. The sequencing results of the screened

| No | Sample Name | Primer | result | Data Group | DNA Sequence |
|---|---|---|---|---|---|
| 1 | 191 | phagemidprimer | GOOD | S | GCAGCACAACTAGCAAGAAGAACAAGCTGGAGAGCAGCAGCAGAACAAAAACTAATAAGCGAAGAAGACCTAAGCAGATAGGACGACGACGAC |
| 2 | 197 | phagemidprimer | GOOD | S | GCAGCACAACTAGCAAAAAGACTACAAGCAAGAGCAGCAGCAGAACAAAAACTAATAAGCGAAGAAGACCTAAGCAGATAGGACGACGACGAC |
| 3 | 202 | phagemidprimer | GOOD | S | GCAGCACAACTAGCAAAAAGACAATGGGTAAAAGCAGCAGCAGAACAAAAACTAATAAGCGAAGAAGACCTAAGCAGATAGGACGACGACGAC |
| 4 | 221 | phagemidprimer | GOOD | S | GCAGCACAACTAGCAAGAAAAAGACAAACAAGAGCAGCAGCAGAACAAAAACTAATAAGCGAAGAAGACCTAAGCAGATAGGACGACGACGAC |
| 5 | 223 | phagemidprimer | GOOD | S | GCAGCACAACTAGCAAAAAGAAGATTCCAAAAAGCAGCAGCAGAACAAAAACTAATAAGCGAAGAAGACCTAAGCAGATAGGACGACGACGAC |
| 6 | 224 | phagemidprimer | GOOD | S | GCAGCACAACTAGCAAAAAGAGGAAGATGCAAAGCAGCAGCAGAACAAAAACTAATAAGCGAAGAAGACCTAAGCAGATAGGACGACGACGAC |
| 7 | 233 | phagemidprimer | GOOD | S | GCAGCACAACTAGCAAAAAGAAGATTCCAAAAAGCAGCAGCAGAACAAAAACTAATAAGCGAAGAAGACCTAAGCAGATAGGACGACGACGAC |
| 8 | 289 | phagemidprimer | GOOD | S | GCAGCACAACTAGCAAGAAGAGGAGTAAGAAGAGCAGCAGCAGAACAAAAACTAATAAGCGAAGAAGACCTAAGCAGATAGGACGACGACGAC |
| 9 | 290 | phagemidprimer | GOOD | S | GCAGCACAACTAGCAAGAAAAAGAATAAGAAGAGCAGCAGCAGAACAAAAACTAATAAGCGAAGAAGACCTAAGCAGATAGGACGACGACGAC |

Supplementary Table S2. Skin irritation index of a novel peptide (peptide 289, Medipep)

| Novel peptide | 30 min | | | | 24 hours | | | | Average response (%) | |
|---|---|---|---|---|---|---|---|---|---|---|
| | ± | 1+ | 2+ | 3+ | ± | 1+ | 2+ | 3+ | | |
| 0.1ppm | 0 | 0 | 0 | 0 | 0 | 0 | 0 | 0 | 0.0±0.00 | No irritation |
| 1ppm | 0 | 0 | 0 | 0 | 0 | 0 | 0 | 0 | 0.0±0.00 | No irritation |
| 10ppm | 0 | 0 | 0 | 0 | 0 | 0 | 0 | 0 | 0.0±0.00 | No irritation |
| 100ppm | 0 | 0 | 0 | 0 | 0 | 0 | 0 | 0 | 0.0±0.00 | No irritation |

Supplementary Table S3. Eye irritation index of a novel peptide (peptide 289, Medipep)

| Sample | Concentration | Absorption | | Average absorption | | Cell survival (%Control) | Standard |
|---|---|---|---|---|---|---|---|
| | | (N1) | (N2) | | | | |
| Control (DPBS) | - | 1.448 | 1.45 | 1.449 | 1.395 | 100.00% | Non-Irritant |
| Methyl acetate | - | 0.102 | 0.138 | 0.12 | 0.066 | 4.70% | Irritant |
| Novel peptide | 0.1ppm | 1.461 | 1.585 | 1.523 | 1.469 | 105.30% | Non-Irritant |
| | 1ppm | 1.558 | 1.656 | 1.607 | 1.553 | 111.30% | Non-Irritant |
| | 10ppm | 1.464 | 1.583 | 1.524 | 1.47 | 105.30% | Non-Irritant |
| | 100ppm | 1.563 | 1.456 | 1.51 | 1.456 | 104.30% | Non-Irritant |

Supplementary Table S4. All primers for the quantitative reverse transcription PCR (qRT-PCR)

| Target | Primer | Sequences |
|---|---|---|
| Human *AQP3* | Forward | 5′-ACCTTTGCCATGTGCTTCCT-3′ |
| | Reverse | 5′-GCGTCTGTGCCAGGGTGTA-3′ |
| Human *HAS-2* | Forward | 5′-CCCAAAATGTGAAGCTTGGT-3′ |
| | Reverse | 5′-CAGGCCACAGAACAAAACCT-3′ |
| Human *GAPDH* | Forward | 5′-TGCACCACCAACTGCTTAGC-3′ |
| | Reverse | 5′-GGCATGGACTGTGGTCATGAG-3′ |

The Mass spectrometry and chromatography data of the synthesized peptides list.

| Peptide number | Sequence | Lot number | Product name |
|---|---|---|---|
| 289 | RRGVRR | K201483 | P1H-6mer |
| 290 | RKRIRR | K201486 | L3-28_6mer |
| 224 | KRGRCK | K201485 | L3-37_6mer |
| 221 | RKRQTR | K201484 | 110_6mer |
| 223 | KRRFQK | K202196 | 2_6m_5_41 |
| 202 | KRQWVK | K202193 | 2_6m_5_33 |
| 191 | RRTSWR | K202194 | 2_6m_5_34 |
| 197 | KRLQAR | K202192 | 2_6m_5_11 |
| 233 | RRQTHK | K202195 | 2_6m_5_35 |

# Certificate of Analysis

| | | |
|---|---|---|
| Lot number | K202194 | |
| Product | 2_6m_5_34 | |
| Sequence | RRTSWR | |
| Appearance | White amorphous powder | |
| Quantity ordered | 1.0 mg | |
| Quantity delivered | 1.3 mg | |
| HPLC analysis | Purity | 96.5% |
| | Instrument | Shimadzu HPLC LabSolution |
| | Column | YMC-Triart C18 /S-5 μm /12nm. |
| | Gradient | 0-60% B Buffer in 30min. |
| | Buffer | A Buffer : 0.2% TFA/$H_2O$ |
| | | B Buffer : 0.2% TFA/Acetonitrile |
| | Flow rate | 1 ml/min. |
| | Wavelength | 230 nm |
| | Temperature | 35 ℃ |
| | Injection volume | 10 μl (0.5 mg/ml) |
| Solubility | Soluble in water | 1.0 mg/ml |
| Mass analysis | Instrument | AXIMA Assurance, MALDI-TOF, Shimadzu |
| | MS expected | 861.0 Da |
| | MS found | 861.5 Da |

Remarks

1. Not for Human Use. Research Purposes Only.
2. This peptide contains unspecified amount of trifluoroacetic acid(TFA) unless exchanged with other type of salts.
3. Due to unknown stability of this peptide, it is highly recommended that the peptide be resolved in medium right prior to use.

Released by J.E. Kang Date 2020 . 9 . 23

Rm.206, Pilot plant , Gwangju Technopark,
Cheomdankwagiro 333, Buk-gu, Gwang-ju, 61008, Korea
T: +82-62-714-1166 F: +82-62-714-1188
E-mail : order@anygen.com

SHIMADZU LabSolutions

# Analysis Report

## <Sample Information>

Sample Name :K202194 (2_6m_5_34)

## <Chromatogram>

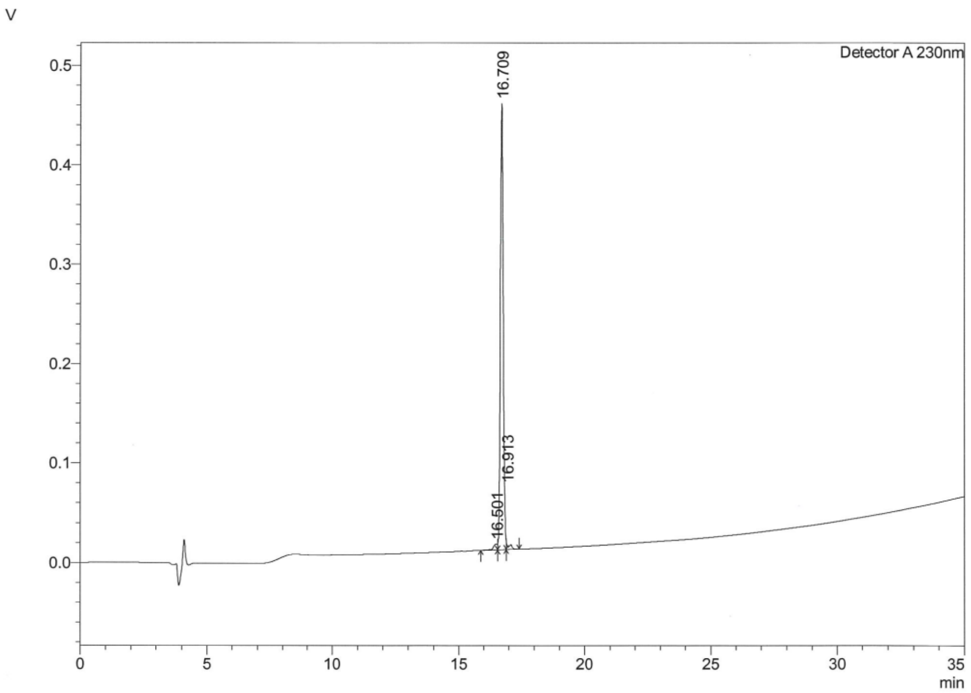


## <Peak Table>

Detector A 230nm

| Peak# | Ret. Time | Area | Area% |
|---|---|---|---|
| 1 | 16.501 | 71699 | 1.926 |
| 2 | 16.709 | 3592297 | 96.507 |
| 3 | 16.913 | 58309 | 1.566 |
| Total | | 3722304 | 100.000 |

HPLC : 20A

ANYGEN
K202194
Data: <Untitled>.J1[c] 18 Sep 2020 11:14 Cal: 7 Apr 2015 20:50
Shimadzu Biotech Axima Assurance 2.9.3.20110624: Mode Linear_20190614, Power: 24, P.Ext. @ 4000 (bin 80)
%Int. 41 mV Profiles 1-36: Threshold 25% Centroid [Adaptive]

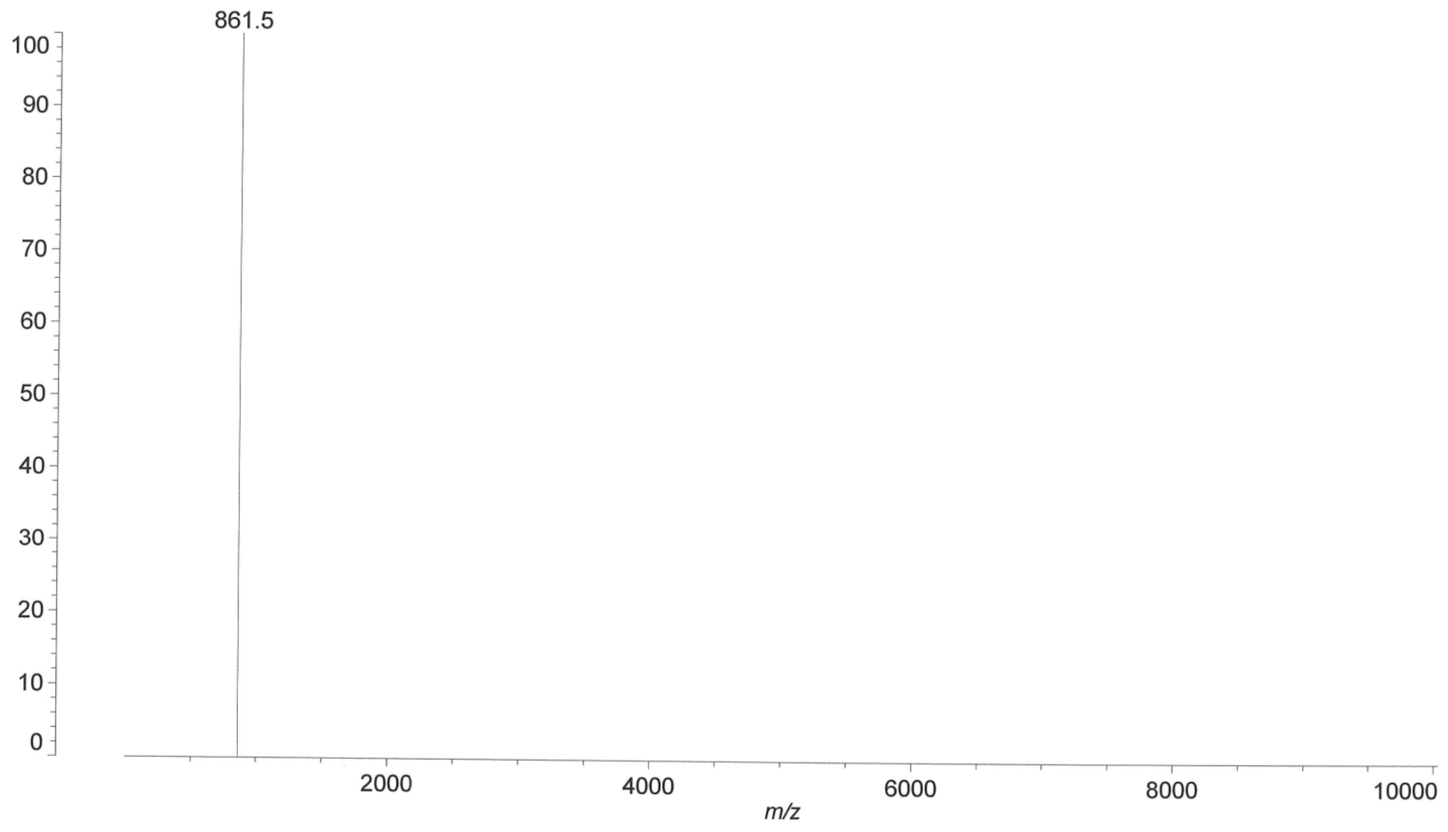

# Certificate of Analysis

ANYGEN

| | | |
|---|---|---|
| Lot number | K202192 | |
| Product | 2_6m_5_11 | |
| Sequence | KRLQAR | |
| Appearance | White amorphous powder | |
| Quantity ordered | 1.0 mg | |
| Quantity delivered | 1.1 mg | |
| HPLC analysis | Purity | 90.3% |
| | Instrument | Shimadzu HPLC LabSolution |
| | Column | YMC-Triart C18 /S-5 µm /12nm. |
| | Gradient | 0-60% B Buffer in 30min. |
| | Buffer | A Buffer : 0.2% TFA/$H_2O$ |
| | | B Buffer : 0.2% TFA/Acetonitrile |
| | Flow rate | 1 ml/min. |
| | Wavelength | 230 nm |
| | Temperature | 35 ℃ |
| | Injection volume | 100 µl (0.5 mg/ml) |
| Solubility | Soluble in water | 1.0 mg/ml |
| Mass analysis | Instrument | AXIMA Assurance, MALDI-TOF, Shimadzu |
| | MS expected | 770.9 Da |
| | MS found | 770.7 Da |

Remarks

1. Not for Human Use. Research Purposes Only.
2. This peptide contains unspecified amount of trifluoroacetic acid(TFA) unless exchanged with other type of salts.
3. Due to unknown stability of this peptide, it is highly recommended that the peptide be resolved in medium right prior to use.

Released by J.E. Kang Date 2020 . 9 . 23

Rm.206, Pilot plant , Gwangju Technopark,
Cheomdankwagiro 333, Buk-gu, Gwang-ju, 61008, Korea
T: +82-62-714-1166 F: +82-62-714-1188
E-mail : order@anygen.com

# Analysis Report

SHIMADZU LabSolutions

## <Sample Information>

Sample Name :K202192 (2_6m_5_11)

## <Chromatogram>

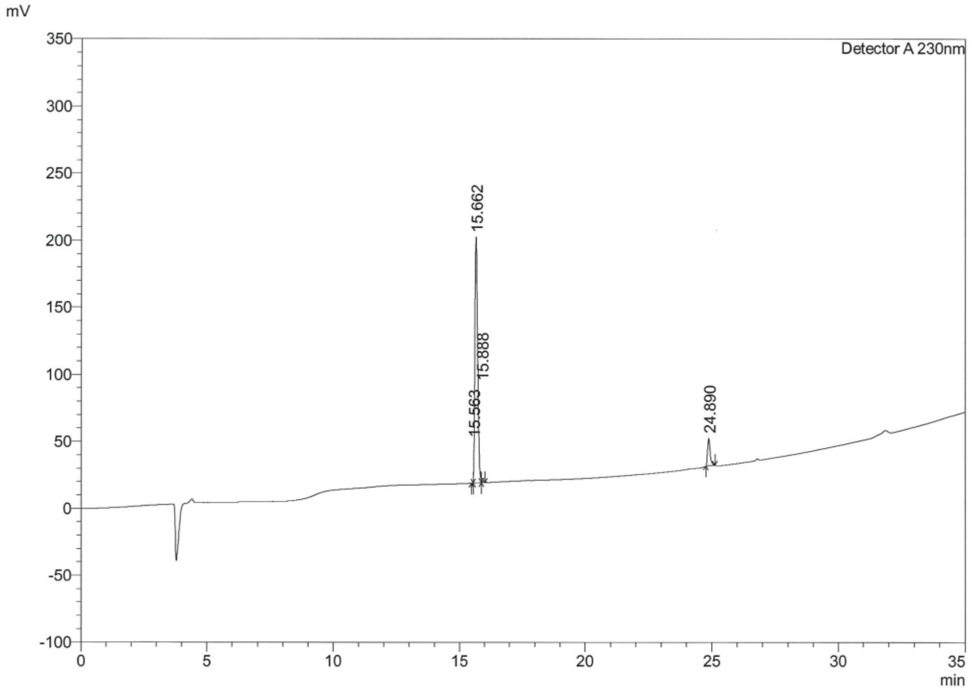


## <Peak Table>

Detector A 230nm

| Peak# | Ret. Time | Area | Area% |
|---|---|---|---|
| 1 | 15.563 | 3722 | 0.236 |
| 2 | 15.662 | 1425446 | 90.301 |
| 3 | 15.888 | 3532 | 0.224 |
| 4 | 24.890 | 145858 | 9.240 |
| Total | | 1578557 | 100.000 |

HPLC : 20A

ANYGEN
K202192
Data: <Untitled>.H1[c] 18 Sep 2020 11:14 Cal: 7 Apr 2015 20:50
Shimadzu Biotech Axima Assurance 2.9.3.20110624: Mode Linear_20190614, Power: 24, P.Ext. @ 4000 (bin 80)
%Int. 5.43 mV Profiles 1-19: Threshold 25% Centroid [Adaptive]

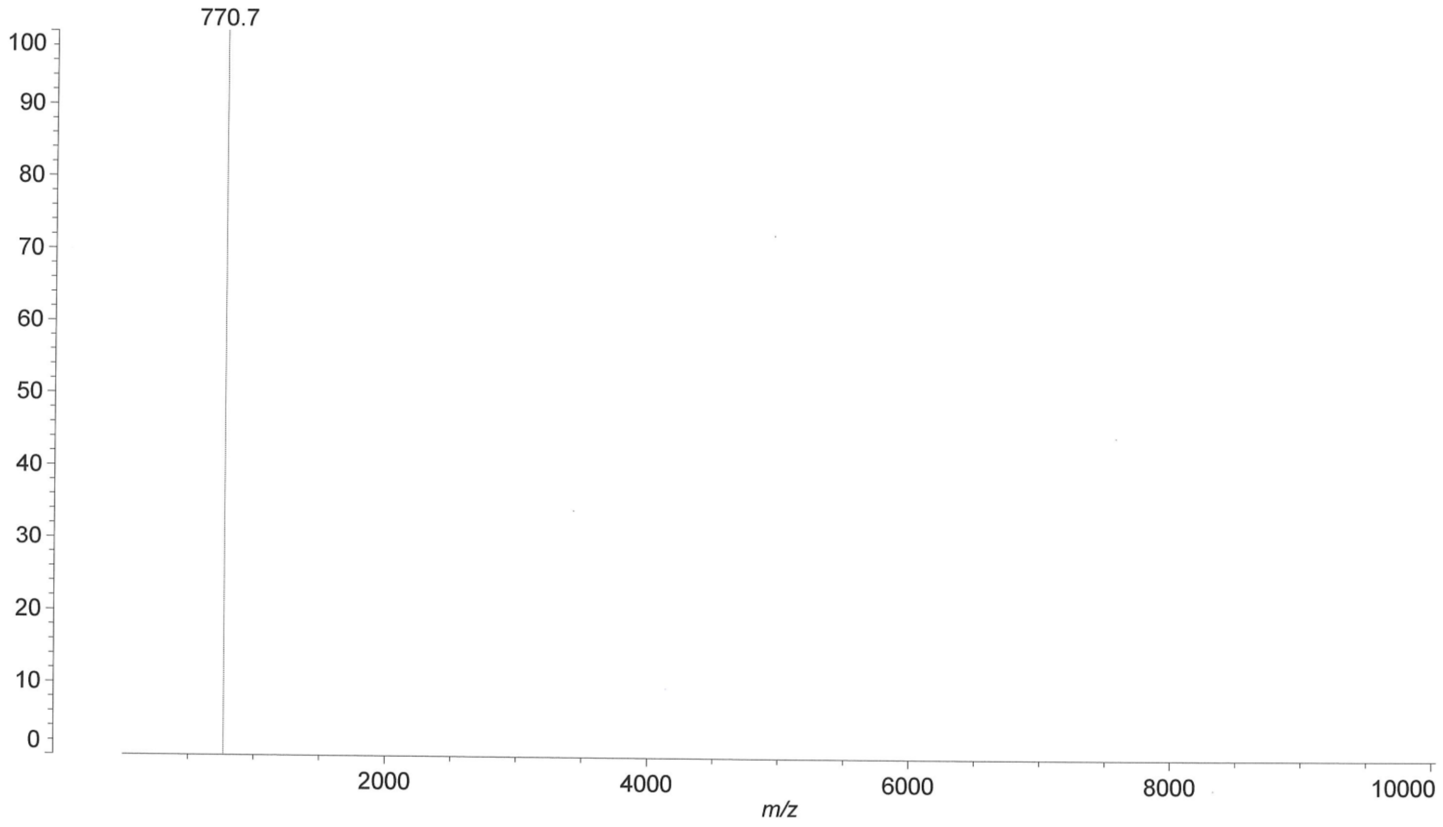

# Certificate of Analysis

| | | |
|---|---|---|
| Lot number | K202193 | |
| Product | 2_6m_5_33 | |
| Sequence | KRQWVK | |
| Appearance | White amorphous powder | |
| Quantity ordered | 1.0 mg | |
| Quantity delivered | 1.3 mg | |
| HPLC analysis | Purity | 98.4% |
| | Instrument | Shimadzu HPLC LabSolution |
| | Column | YMC-Triart C18 /S-5 µm /12nm. |
| | Gradient | 0-60% B Buffer in 30min. |
| | Buffer | A Buffer : 0.2% TFA/$H_2O$ |
| | | B Buffer : 0.2% TFA/Acetonitrile |
| | Flow rate | 1 ml/min. |
| | Wavelength | 230 nm |
| | Temperature | 35 °C |
| | Injection volume | 10 µl (0.5 mg/ml) |
| Solubility | Soluble in water | 1.0 mg/ml |
| Mass analysis | Instrument | AXIMA Assurance, MALDI-TOF, Shimadzu |
| | MS expected | 844.0 Da |
| | MS found | 844.1 Da |

Remarks

1. Not for Human Use. Research Purposes Only.
2. This peptide contains unspecified amount of trifluoroacetic acid(TFA) unless exchanged with other type of salts.
3. Due to unknown stability of this peptide, it is highly recommended that the peptide be resolved in medium right prior to use.

Released by J.E. Kang Date 2020 . 9 . 23

Rm.206, Pilot plant , Gwangju Technopark,
Cheomdankwagiro 333, Buk-gu, Gwang-ju, 61008, Korea
T: +82-62-714-1166 F: +82-62-714-1188
E-mail : order@anygen.com

SHIMADZU LabSolutions

# Analysis Report

## <Sample Information>

Sample Name :K202193 (2_6m_5_33)

## <Chromatogram>

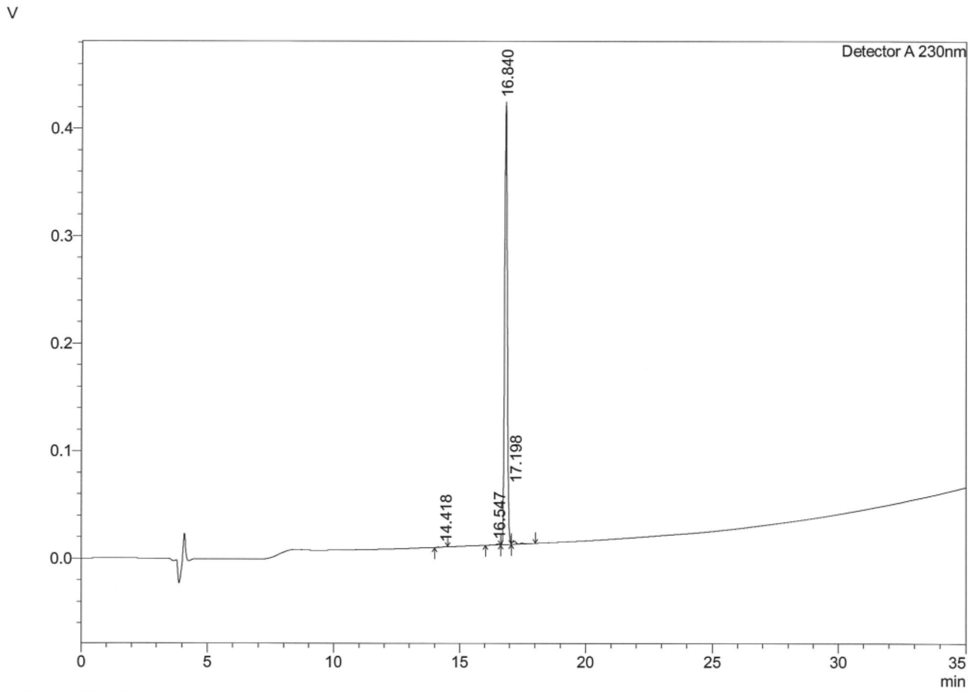


## <Peak Table>

Detector A 230nm

| Peak# | Ret. Time | Area | Area% |
|---|---|---|---|
| 1 | 14.418 | 4459 | 0.144 |
| 2 | 16.547 | 9822 | 0.316 |
| 3 | 16.840 | 3057220 | 98.405 |
| 4 | 17.198 | 35280 | 1.136 |
| Total | | 3106780 | 100.000 |

HPLC : 20A

ANYGEN
K202193
Data: <Untitled>.I1[c] 18 Sep 2020 11:14 Cal: 7 Apr 2015 20:50
Shimadzu Biotech Axima Assurance 2.9.3.20110624: Mode Linear_20190614, Power: 24, P.Ext. @ 4000 (bin 80)
%Int. 45 mV Profiles 1-23: Threshold 25% Centroid [Adaptive]

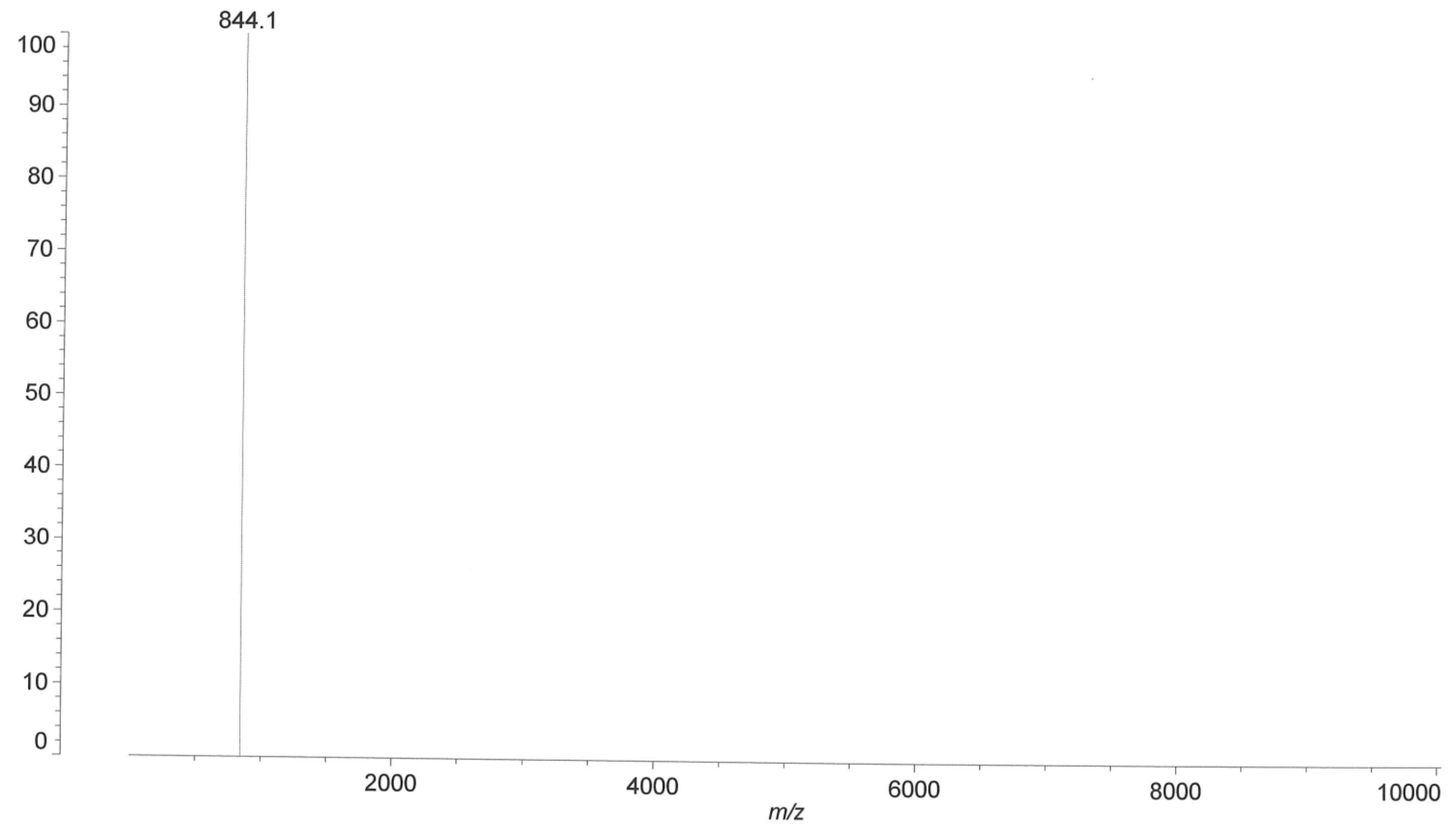

# Certificate of Analysis

ANYGEN

| | | |
|---|---|---|
| Lot number | K201484 | |
| Product | 110_6mer | |
| Sequence | RKRQTR | |
| Appearance | White amorphous powder | |
| Quantity ordered | 1.0 mg | |
| Quantity delivered | 1.8 mg | |
| HPLC analysis | Purity | 90.1% |
| | Instrument | Shimadzu HPLC LabSolution |
| | Column | YMC-Triart C18 /S-5μm /12nm. |
| | Gradient | 0-60% B Buffer in 30min. |
| | Buffer | A Buffer : 0.2% TFA/$H_2O$ |
| | | B Buffer : 0.2% TFA/Acetonitrile |
| | Flow rate | 1 ml/min. |
| | Wavelength | 230 nm |
| | Temperature | 35 ℃ |
| | Injection volume | 20 μl (0.5 mg/ml) |
| Solubility | Soluble in water | 1.0 mg/ml |
| Mass analysis | Instrument | AXIMA Assurance, MALDI-TOF, Shimadzu |
| | MS expected | 844.0 Da |
| | MS found | 844.8 Da |

Remarks

1. Not for Human Use. Research Purposes Only.
2. This peptide contains unspecified amount of trifluoroacetic acid(TFA) unless exchanged with other type of salts.
3. Due to unknown stability of this peptide, it is highly recommended that the peptide be resolved in medium right prior to use.

Released by K.J. Lee Date 2020 . 7 . 16

Rm.206, Pilot plant , Gwangju Technopark,
Cheomdankwagiro 333, Buk-gu, Gwang-ju, 61008, Korea
T: +82-62-714-1166 F: +82-62-714-1188
E-mail : order@anygen.com

SHIMADZU LabSolutions

# Analysis Report

## <Sample Information>

Sample Name :K201484 (110_6mer)

## <Chromatogram>

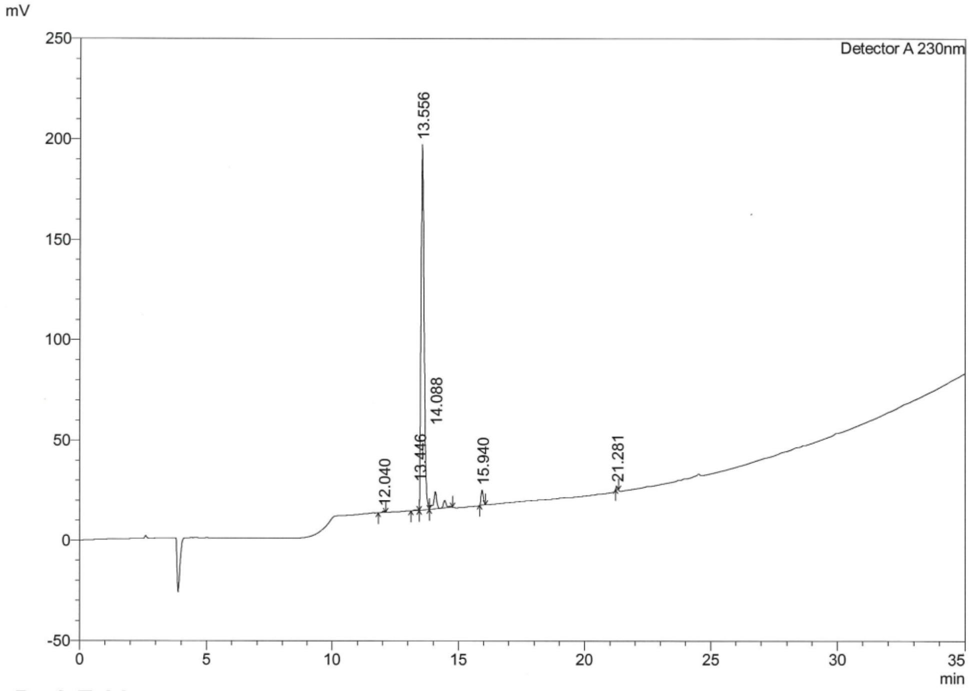


## <Peak Table>

Detector A 230nm

| Peak# | Ret. Time | Area | Area% |
|---|---|---|---|
| 1 | 12.040 | 3911 | 0.230 |
| 2 | 13.446 | 3817 | 0.225 |
| 3 | 13.556 | 1531904 | 90.135 |
| 4 | 14.088 | 106776 | 6.283 |
| 5 | 15.940 | 46948 | 2.762 |
| 6 | 21.281 | 6203 | 0.365 |
| Total | | 1699557 | 100.000 |

HPLC : 20A

ANYGEN
K201484
Data: <Untitled>.L14[c] 8 Jul 2020 12:38 Cal: 7 Apr 2015 20:50
Shimadzu Biotech Axima Assurance 2.9.3.20110624: Mode Linear_20190614, Power: 30, P.Ext. @ 1000 (bin 56)
%Int. 203 mV Profiles 22-104: Threshold 25% Centroid [Adaptive]

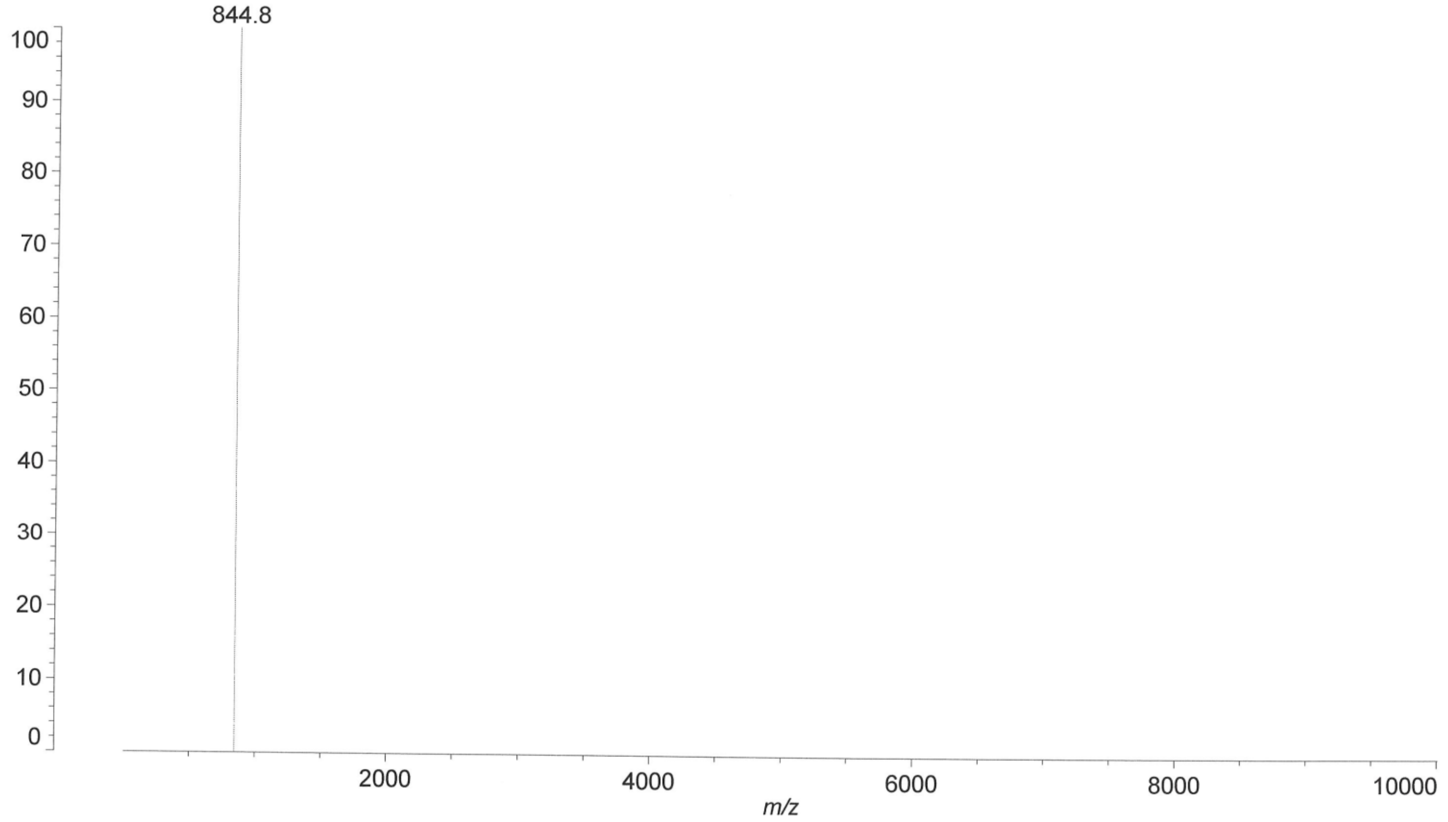

# Certificate of Analysis

| | | |
|---|---|---|
| Lot number | K202196 | |
| Product | 2_6m_5_41 | |
| Sequence | KRRFQK | |
| Appearance | White amorphous powder | |
| Quantity ordered | 1.0 mg | |
| Quantity delivered | 1.2 mg | |
| HPLC analysis | Purity | 97.3% |
| | Instrument | Shimadzu HPLC LabSolution |
| | Column | YMC-Triart C18 /S-5 μm /12nm. |
| | Gradient | 0-60% B Buffer in 30min. |
| | Buffer | A Buffer : 0.2% TFA/$H_2O$ |
| | | B Buffer : 0.2% TFA/Acetonitrile |
| | Flow rate | 1 ml/min. |
| | Wavelength | 230 nm |
| | Temperature | 35 ℃ |
| | Injection volume | 100 μl (0.5 mg/ml) |
| Solubility | Soluble in water | 1.0 mg/ml |
| Mass analysis | Instrument | AXIMA Assurance, MALDI-TOF, Shimadzu |
| | MS expected | 862.0 Da |
| | MS found | 862.5 Da |

Remarks

1. Not for Human Use. Research Purposes Only.
2. This peptide contains unspecified amount of trifluoroacetic acid(TFA) unless exchanged with other type of salts.
3. Due to unknown stability of this peptide, it is highly recommended that the peptide be resolved in medium right prior to use.

Released by J.E. Kang Date 2020 . 9 . 23

Rm.206, Pilot plant , Gwangju Technopark,
Cheomdankwagiro 333, Buk-gu, Gwang-ju, 61008, Korea
T: +82-62-714-1166 F: +82-62-714-1188
E-mail : order@anygen.com

www.anygen.com

SHIMADZU LabSolutions

# Analysis Report

## <Sample Information>

Sample Name :K202196 (2_6m_5_41)

## <Chromatogram>

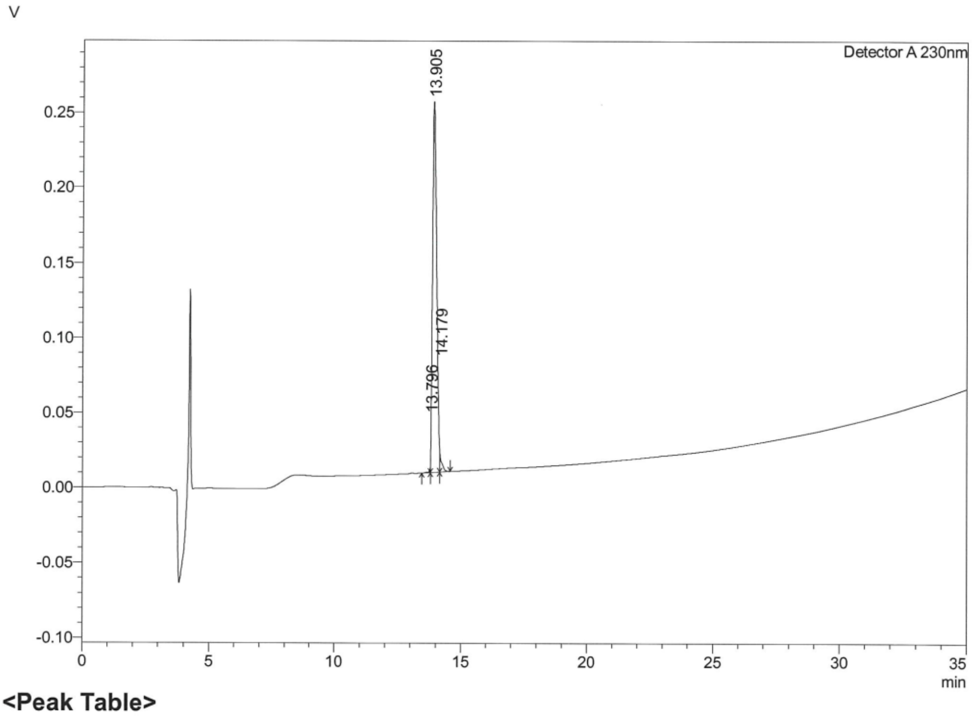


## <Peak Table>

Detector A 230nm

| Peak# | Ret. Time | Area | Area% |
|---|---|---|---|
| 1 | 13.796 | 11111 | 0.392 |
| 2 | 13.905 | 2758128 | 97.335 |
| 3 | 14.179 | 64391 | 2.272 |
| Total | | 2833630 | 100.000 |

HPLC : 20A

ANYGEN
K202196
Data: <Untitled>.L1[c] 18 Sep 2020 11:16 Cal: 7 Apr 2015 20:50
Shimadzu Biotech Axima Assurance 2.9.3.20110624: Mode Linear_20190614, Power: 25, P.Ext. @ 4000 (bin 80)
%Int. 167 mV Profiles 1-43: Threshold 25% Centroid [Adaptive]

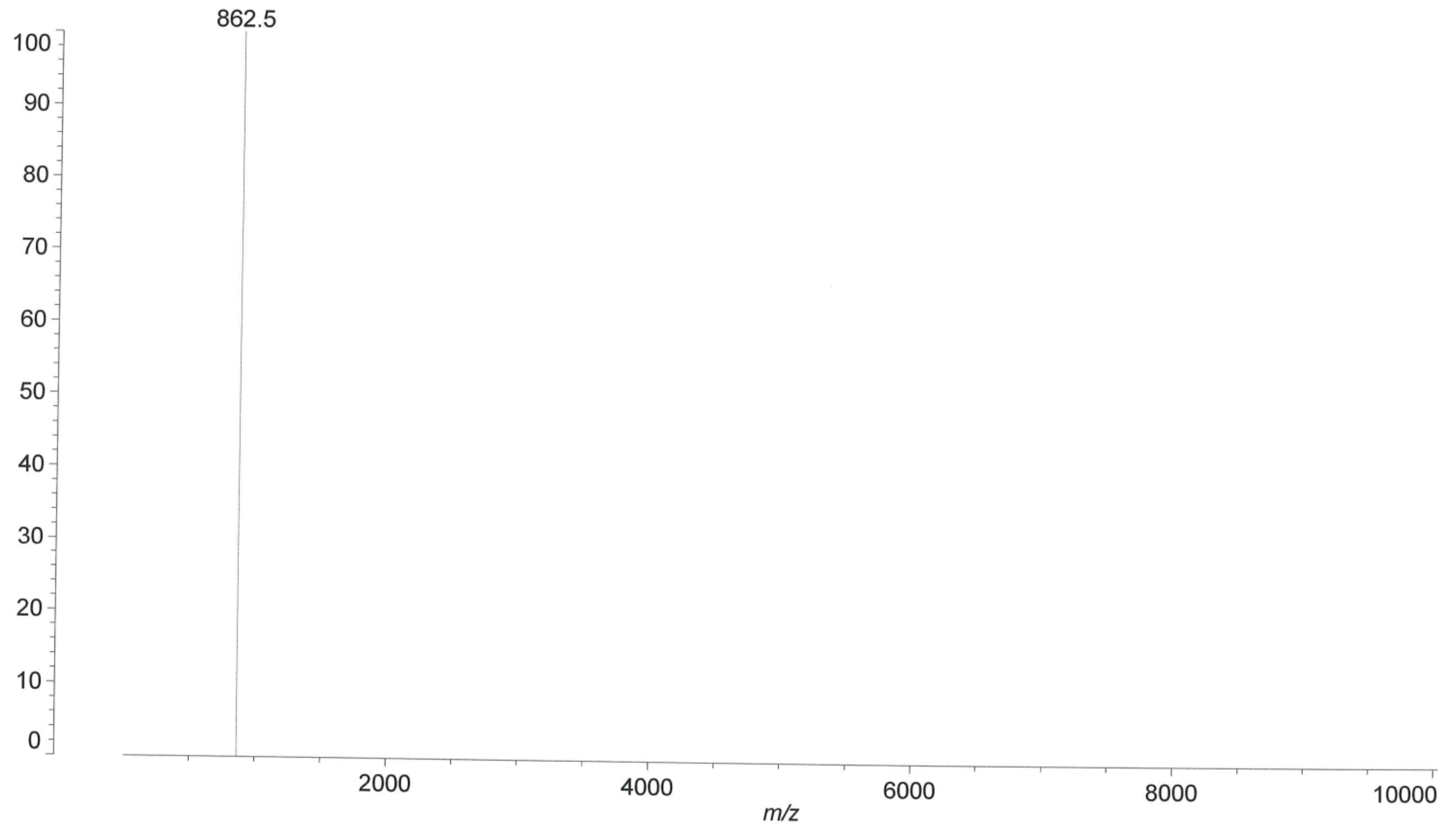

# Certificate of Analysis

| | | |
|---|---|---|
| Lot number | K201485 | |
| Product | L3-37_6mer | |
| Sequence | KRGRCK | |
| Appearance | White amorphous powder | |
| Quantity ordered | 1.0 mg | |
| Quantity delivered | 1.7 mg | |
| HPLC analysis | Purity | 90.0% |
| | Instrument | Shimadzu HPLC LabSolution |
| | Column | YMC-Triart C18 /S-5μm /12nm. |
| | Gradient | 0-60% B Buffer in 30min. |
| | Buffer | A Buffer : 0.2% TFA/$H_2O$ |
| | | B Buffer : 0.2% TFA/Acetonitrile |
| | Flow rate | 1 ml/min. |
| | Wavelength | 230 nm |
| | Temperature | 35 ℃ |
| | Injection volume | 20 μl (0.5 mg/ml) |
| Solubility | Soluble in water | 1.0 mg/ml |
| Mass analysis | Instrument | AXIMA Assurance, MALDI-TOF, Shimadzu |
| | MS expected | 746.9 Da |
| | MS found | 746.9 Da |

Remarks
1. Not for Human Use. Research Purposes Only.
2. This peptide contains unspecified amount of trifluoroacetic acid(TFA) unless exchanged with other type of salts.
3. Due to unknown stability of this peptide, it is highly recommended that the peptide be resolved in medium right prior to use.

Released by K.J. Lee Date 2020 . 7 . 16

Rm.206, Pilot plant , Gwangju Technopark,
Cheomdankwagiro 333, Buk-gu, Gwang-ju, 61008, Korea
T: +82-62-714-1166 F: +82-62-714-1188
E-mail : order@anygen.com

SHIMADZU LabSolutions

# Analysis Report

## <Sample Information>

Sample Name :K201485 (L3-37_6mer)

## <Chromatogram>

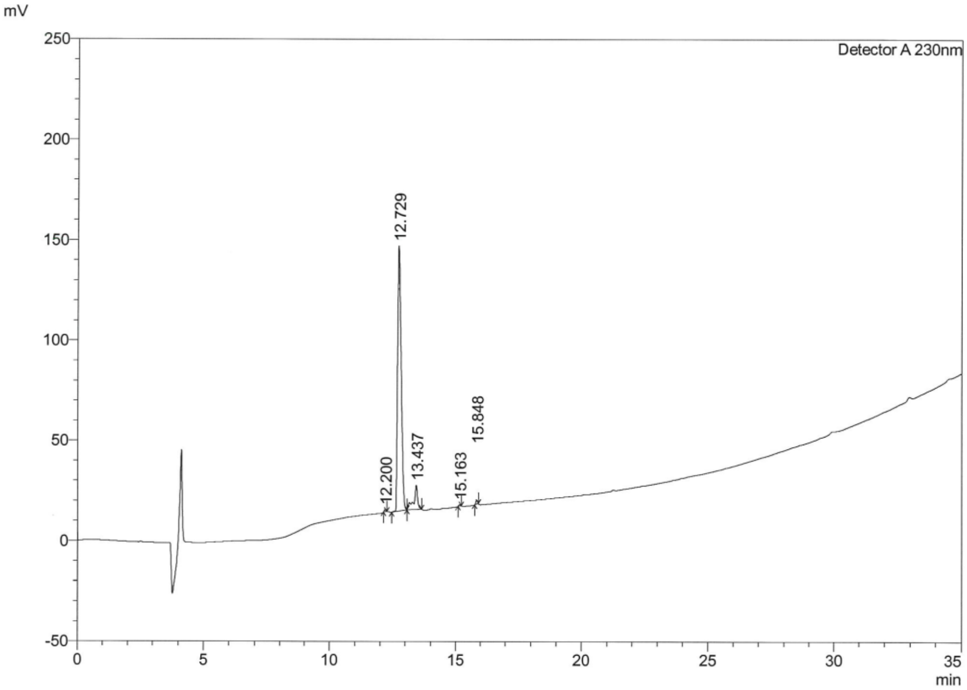


## <Peak Table>

Detector A 230nm

| Peak# | Ret. Time | Area | Area% |
|---|---|---|---|
| 1 | 12.200 | 4888 | 0.335 |
| 2 | 12.729 | 1312189 | 90.013 |
| 3 | 13.437 | 128010 | 8.781 |
| 4 | 15.163 | 3549 | 0.243 |
| 5 | 15.848 | 9143 | 0.627 |
| Total | | 1457779 | 100.000 |

HPLC : 20A

ANYGEN
K201485
Data: <Untitled>.G6[c] 16 Jul 2020 14:44 Cal: peptide
Shimadzu Biotech Axima Assurance 2.9.3.20110624: Mode Linear_20190614, Power: 36, P.Ext. @ 3000 (bin 69)
%Int. 120 mV Profiles 1-29: Threshold 25% Centroid [Adaptive]

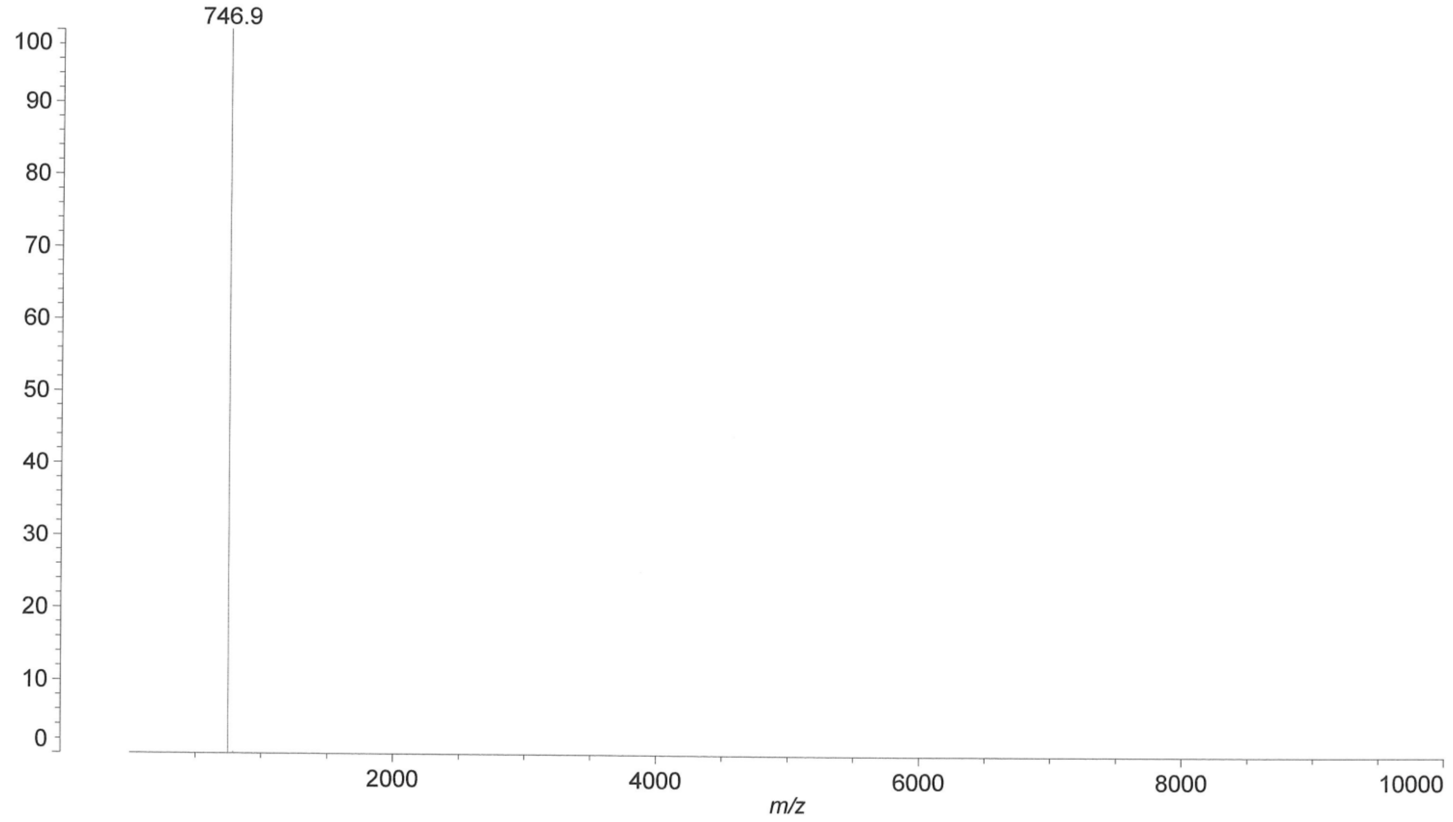

# Certificate of Analysis

ANYGEN

| | | |
|---|---|---|
| Lot number | K202195 | |
| Product | 2_6m_5_35 | |
| Sequence | RRQTHK | |
| Appearance | White amorphous powder | |
| Quantity ordered | 1.0 mg | |
| Quantity delivered | 2.1 mg | |
| HPLC analysis | Purity | 99.8% |
| | Instrument | Shimadzu HPLC LabSolution |
| | Column | YMC-Triart C18 /S-5 μm /12nm. |
| | Gradient | 0-60% B Buffer in 30min. |
| | Buffer | A Buffer : 0.2% TFA/$H_2O$ |
| | | B Buffer : 0.2% TFA/Acetonitrile |
| | Flow rate | 1 ml/min. |
| | Wavelength | 230 nm |
| | Temperature | 35 ℃ |
| | Injection volume | 100 μl (0.5 mg/ml) |
| Solubility | Soluble in water | 1.0 mg/ml |
| Mass analysis | Instrument | AXIMA Assurance, MALDI-TOF, Shimadzu |
| | MS expected | 824.9 Da |
| | MS found | 825.5 Da |

Remarks

1. Not for Human Use. Research Purposes Only.
2. This peptide contains unspecified amount of trifluoroacetic acid(TFA) unless exchanged with other type of salts.
3. Due to unknown stability of this peptide, it is highly recommended that the peptide be resolved in medium right prior to use.

Released by J.E. Kang Date 2020. 9. 23

Rm.206, Pilot plant , Gwangju Technopark,
Cheomdankwagiro 333, Buk-gu, Gwang-ju, 61008, Korea
T: +82-62-714-1166 F: +82-62-714-1188
E-mail : order@anygen.com

SHIMADZU LabSolutions

# Analysis Report

## <Sample Information>

Sample Name :K202195 (2_6m_5_35)

## <Chromatogram>

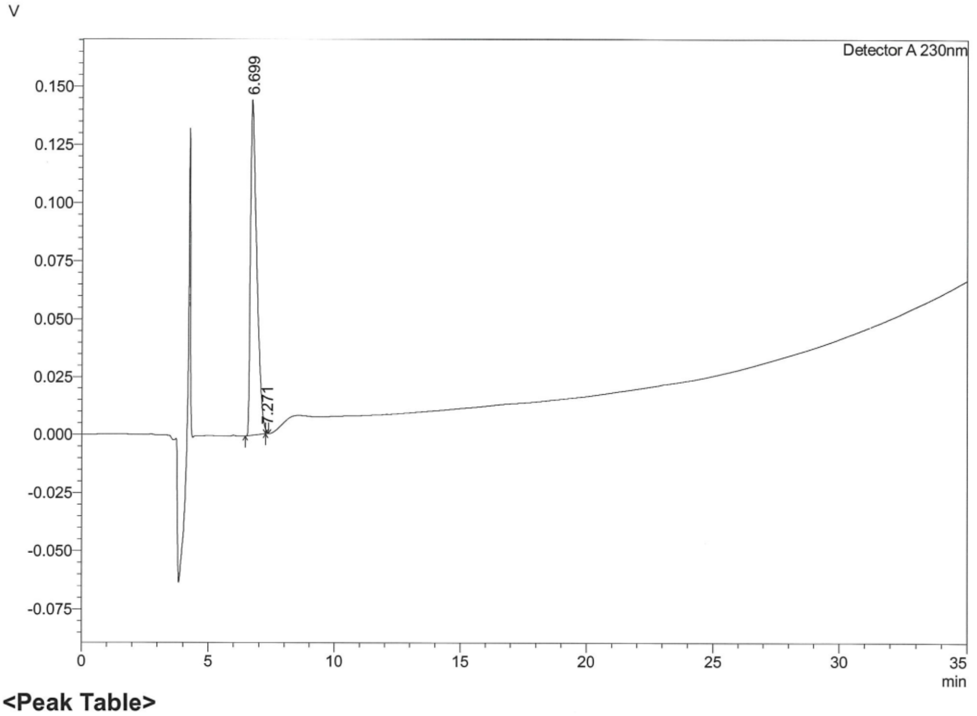


## <Peak Table>

Detector A 230nm

| Peak# | Ret. Time | Area | Area% |
|---|---|---|---|
| 1 | 6.699 | 2491905 | 99.854 |
| 2 | 7.271 | 3645 | 0.146 |
| Total | | 2495550 | 100.000 |

HPLC : 20A

ANYGEN
K202195
Data: <Untitled>.K1[c] 18 Sep 2020 11:15 Cal: 7 Apr 2015 20:50
Shimadzu Biotech Axima Assurance 2.9.3.20110624: Mode Linear_20190614, Power: 36, P.Ext. @ 4000 (bin 80)
%Int. 13 mV Profiles 1-29: Threshold 25% Centroid [Adaptive]

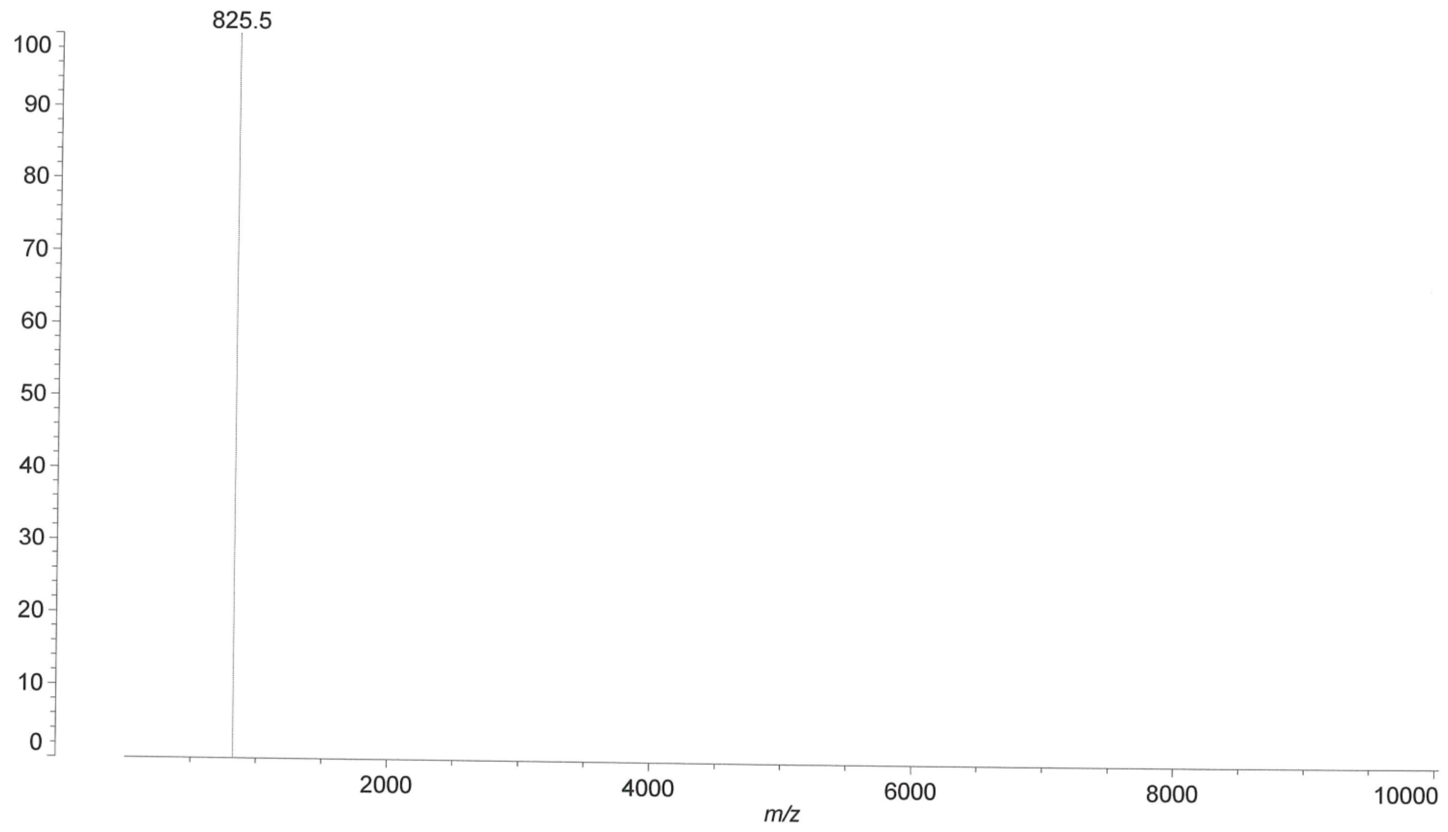

# Certificate of Analysis

ANYGEN

| | | |
|---|---|---|
| Lot number | K201483 | |
| Product | P1H_6mer | |
| Sequence | RRGVRR | |
| Appearance | White amorphous powder | |
| Quantity ordered | 1.0 mg | |
| Quantity delivered | 1.7 mg | |
| HPLC analysis | Purity | 97.0% |
| | Instrument | Shimadzu HPLC LabSolution |
| | Column | YMC-Triart C18 /S-5μm /12nm. |
| | Gradient | 0-60% B Buffer in 30min. |
| | Buffer | A Buffer : 0.2% TFA/$H_2O$ |
| | | B Buffer : 0.2% TFA/Acetonitrile |
| | Flow rate | 1 ml/min. |
| | Wavelength | 230 nm |
| | Temperature | 35 ℃ |
| | Injection volume | 20 μl (0.5 mg/ml) |
| Solubility | Soluble in water | 1.0 mg/ml |
| Mass analysis | Instrument | AXIMA Assurance, MALDI-TOF, Shimadzu |
| | MS expected | 798.9 Da |
| | MS found | 799.0 Da |

Remarks

1. Not for Human Use. Research Purposes Only.
2. This peptide contains unspecified amount of trifluoroacetic acid(TFA) unless exchanged with other type of salts.
3. Due to unknown stability of this peptide, it is highly recommended that the peptide be resolved in medium right prior to use.

Released by K.J. Lee [signature] Date 2020 . 7 . 16

Rm.206, Pilot plant , Gwangju Technopark,
Cheomdankwagiro 333, Buk-gu, Gwang-ju, 61008, Korea
T: +82-62-714-1166 F: +82-62-714-1188
E-mail : order@anygen.com

SHIMADZU LabSolutions

# Analysis Report

## <Sample Information>

Sample Name :K201483 (P1H_6mer)

## <Chromatogram>

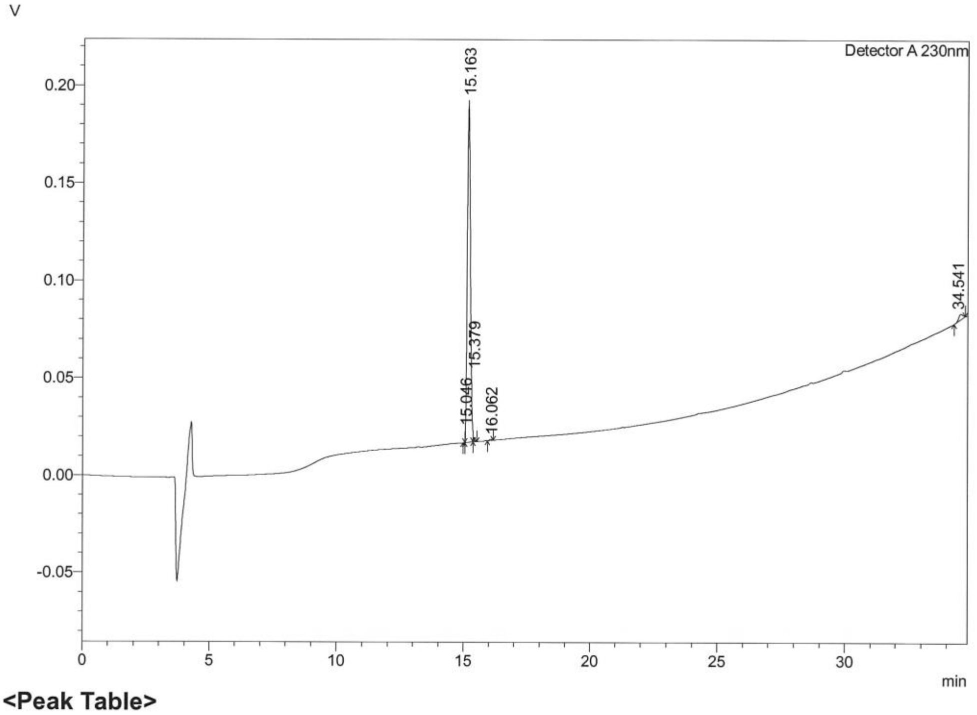


## <Peak Table>

Detector A 230nm

| Peak# | Ret. Time | Area | Area% |
|---|---|---|---|
| 1 | 15.046 | 227 | 0.015 |
| 2 | 15.163 | 1505889 | 97.098 |
| 3 | 15.379 | 7937 | 0.512 |
| 4 | 16.062 | 3566 | 0.230 |
| 5 | 34.541 | 33281 | 2.146 |
| Total | | 1550900 | 100.000 |

HPLC : 20A

ANYGEN
K201483
Data: <Untitled>.F6[c] 16 Jul 2020 14:43 Cal: 7 Apr 2015 20:50
Shimadzu Biotech Axima Assurance 2.9.3.20110624: Mode Linear_20190614, Power: 36, P.Ext. @ 3000 (bin 69)
%Int. 48 mV Profiles 1-49: Threshold 25% Centroid [Adaptive]

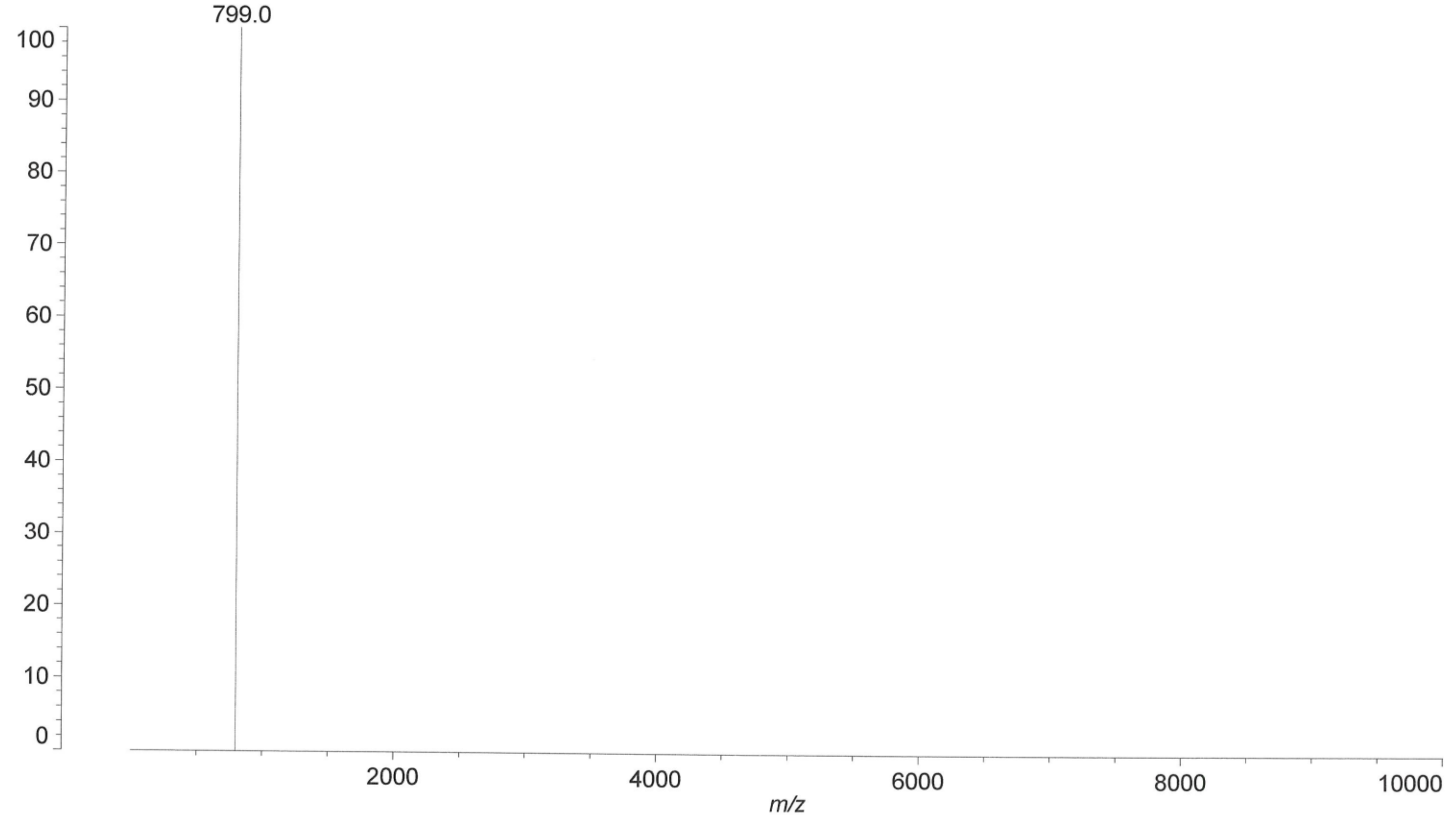

# Certificate of Analysis

| | | |
|---|---|---|
| Lot number | K201486 | |
| Product | L3-28_6mer | |
| Sequence | RKRIRR | |
| Appearance | White amorphous powder | |
| Quantity ordered | 1.0 mg | |
| Quantity delivered | 1.8 mg | |
| HPLC analysis | Purity | 98.7% |
| | Instrument | Shimadzu HPLC LabSolution |
| | Column | YMC-Triart C18 /S-5μm /12nm. |
| | Gradient | 0-60% B Buffer in 30min. |
| | Buffer | A Buffer : 0.2% TFA/$H_2O$ |
| | | B Buffer : 0.2% TFA/Acetonitrile |
| | Flow rate | 1 ml/min. |
| | Wavelength | 230 nm |
| | Temperature | 35 ℃ |
| | Injection volume | 20 μl (0.5 mg/ml) |
| Solubility | Soluble in water | 1.0 mg/ml |
| Mass analysis | Instrument | AXIMA Assurance, MALDI-TOF, Shimadzu |
| | MS expected | 884.1 Da |
| | MS found | 885.0 Da |

Remarks

1. Not for Human Use. Research Purposes Only.
2. This peptide contains unspecified amount of trifluoroacetic acid(TFA) unless exchanged with other type of salts.
3. Due to unknown stability of this peptide, it is highly recommended that the peptide be resolved in medium right prior to use.

Released by K.J. Lee [signature] Date 2020 . 7 . 16

Rm.206, Pilot plant , Gwangju Technopark,
Cheomdankwagiro 333, Buk-gu, Gwang-ju, 61008, Korea
T: +82-62-714-1166 F: +82-62-714-1188
E-mail : order@anygen.com

# SHIMADZU LabSolutions Analysis Report

## <Sample Information>

Sample Name :K201486 (L3-28_6mer)

## <Chromatogram>

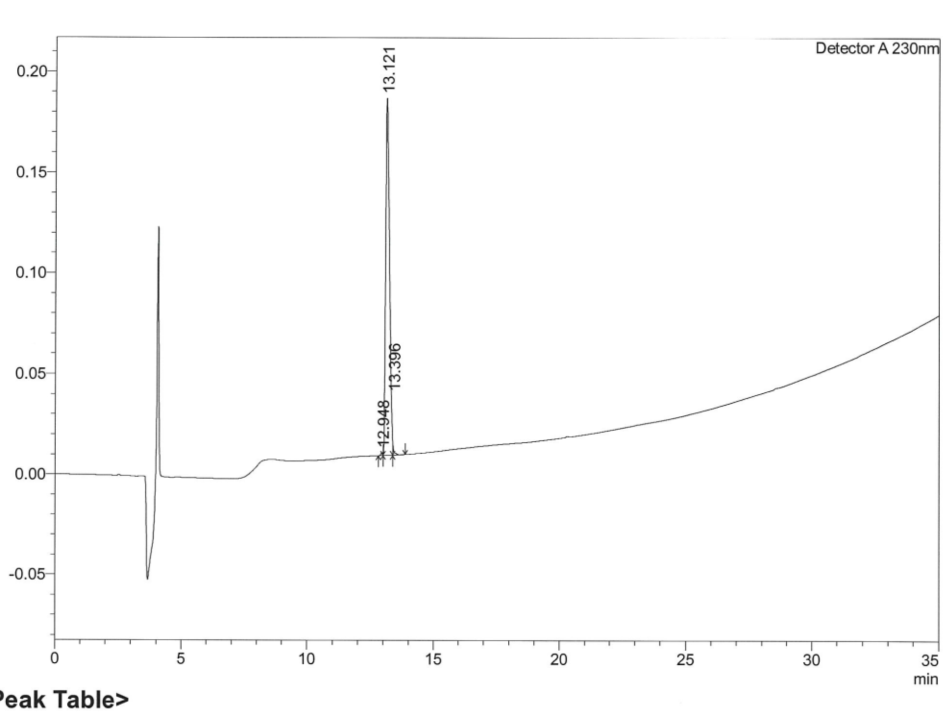


## <Peak Table>

Detector A 230nm

| Peak# | Ret. Time | Area | Area% |
|---|---|---|---|
| 1 | 12.948 | 9484 | 0.495 |
| 2 | 13.121 | 1889435 | 98.706 |
| 3 | 13.396 | 15280 | 0.798 |
| Total | | 1914199 | 100.000 |

HPLC : 20A

ANYGEN
K201486
Data: <Untitled>.N14[c] 8 Jul 2020 12:39 Cal: 7 Apr 2015 20:50
Shimadzu Biotech Axima Assurance 2.9.3.20110624: Mode Linear_20190614, Power: 35, P.Ext. @ 1000 (bin 56)
%Int. 509 mV Profiles 38-110: Threshold 25% Centroid [Adaptive]

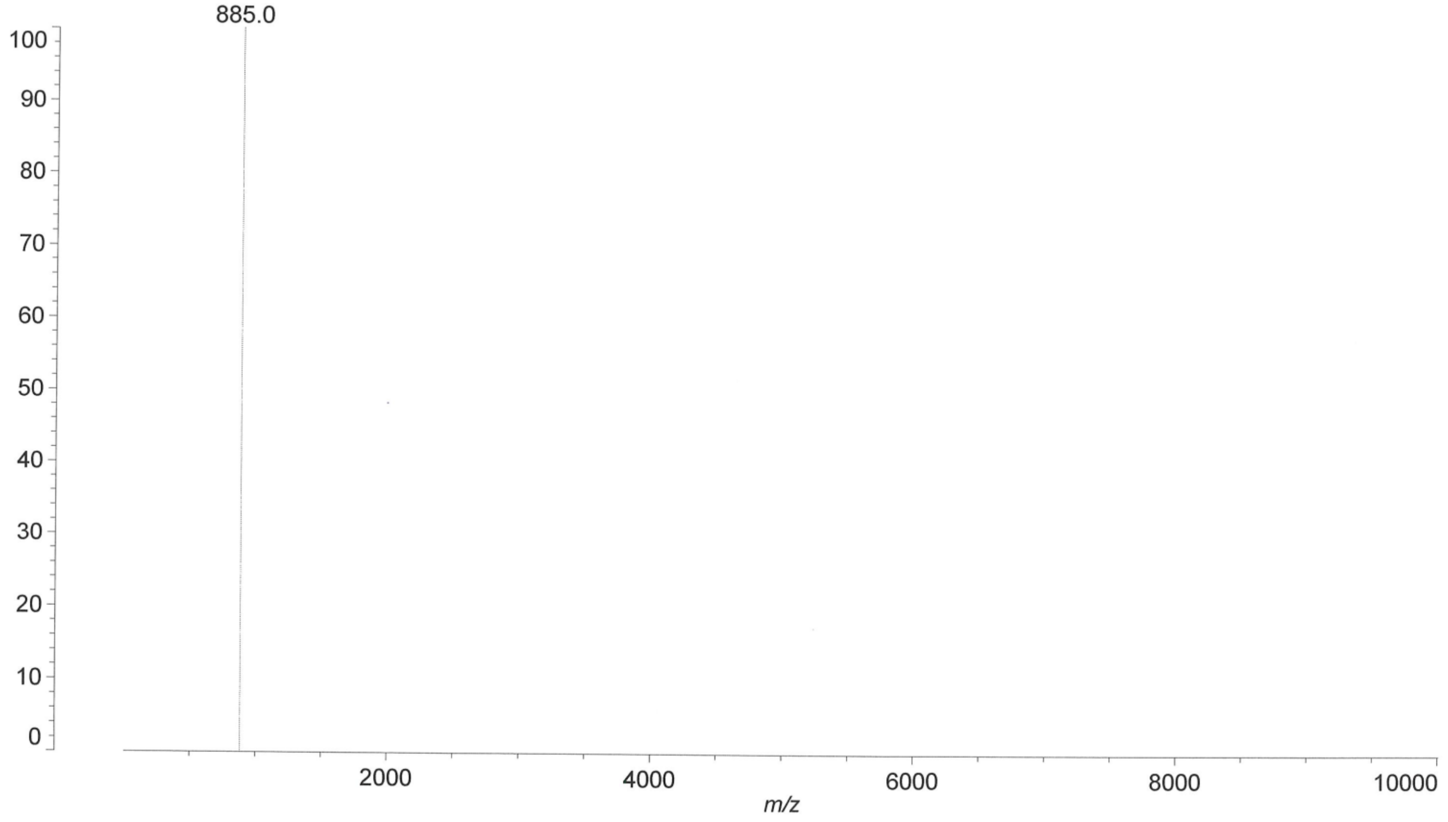

Supplement: Supplementary file 1 [file ijms-25-07860-s001.zip › ijms-3042666-supplementary.pdf]
